# Entropy Sources Based on Silicon Chips: True Random Number Generator and Physical Unclonable Function

**DOI:** 10.3390/e24111566

**Published:** 2022-10-30

**Authors:** Yuan Cao, Wanyi Liu, Lan Qin, Bingqiang Liu, Shuai Chen, Jing Ye, Xianzhao Xia, Chao Wang

**Affiliations:** 1 College of Internet of Things Engineering, Hohai University, Changzhou 213022, China; 2 Rock-Solid Security Lab., Binary Semiconductor Co., Ltd., Suzhou 215000, China; 3 School of Optical and Electronic Information, Huazhong University of Science and Technology, Wuhan 430074, China; 4 Wuhan National Laboratory of Optoelectronics, Huazhong University of Science and Technology, Wuhan 430074, China; 5 State Key Lab of Processors, Institute of Computing Technology, Chinese Academy of Sciences, University of Chinese Academy of Sciences, Beijing 100190, China; 6 China Automotive Technology and Research Center Co., Ltd., Tianjin 300300, China

**Keywords:** entropy, TRNG, PUF, information security

## Abstract

Entropy is a measure of uncertainty or randomness. It is the foundation for almost all cryptographic systems. True random number generators (TRNGs) and physical unclonable functions (PUFs) are the silicon primitives to respectively harvest dynamic and static entropy to generate random bit streams. In this survey paper, we present a systematic and comprehensive review of different state-of-the-art methods to harvest entropy from silicon-based devices, including the implementations, applications, and the security of the designs. Furthermore, we conclude the trends of the entropy source design to point out the current spots of entropy harvesting.

## 1. Introduction

Since the concept of information entropy was introduced by Claude Shannon in 1948 [1], entropy has been widely used in cryptography and cybersecurity. It has been considered as a standard measurement of index to quantize the randomness of secret keys, which are used to protect the sensitive data [2]. The higher the randomness of the keys, the higher the security of the data. There are two types of entropy source, dynamic and static entropy source, which can be generated from silicon chips. Dynamic entropy sources that provide true randomness are usually extracted from the indeterminate physical processes, such as the jitter of ring oscillators (RO) [3,4] and thermal noise from the digital-to-analog converter (DAC) [5], or unpredictable events, such as the human-driven timing of mouse movements and keyboard strokes [6]. Because the indeterminate physical processes are completely determined by the dynamic parameters of the system, this type of entropy is categorized as dynamic entropy. True random number generators (TRNGs) extract the dynamic entropy from random and microscopic fluctuations in physical processes (thermal noise, shot noise, avalanches, clock drift, jitter, atmospheric noise, external electromagnetics, quantum phenomena, etc.), which can generate independent, uniformly distributed, unpredictable random numbers [7,8]. The pseudo random number generator (PRNG) is another type of random number generator, also known as deterministic random bit generator (DRBG), used to produce keys. However, the generated random numbers from PRNG can be predictably traced back to the seed (initial states). In other words, by knowing the seed, it is possible to reconstruct the sequence of numbers produced by a particular PRNG. Therefore, the entropy of the random data generated by PRNG comes from the seed [9]. Strictly speaking, PRNG is an entropy extension and cannot be considered as an entropy source.

The manufacturing variations of nano-scale circuits are generated in the manufacturing process. In other words, static entropy that is extracted from these manufacturing variations are stable once the device is completely fabricated. Physical unclonable function (PUF) takes advantage of this feature to extract the static entropy from uncontrollable and unpredictable variations in the semiconductor manufacturing process and convert it into a binary stream with a predetermined length, which can be used as a key or ID [10,11,12,13,14]. PUF provides a root of trust (RoT) at the hardware layer. Its tamper-resistant property and its ability to securely identify devices by querying, without the need for permanent keys storage or well-defined algorithm support, vastly reduce the risk of many powerful physical attacks, such as reverse engineering, probing, fault injection attacks, security tokens, etc. [15,16,17,18].

Complementary metal-oxide-semiconductor (CMOS)-technology-based designs are advantageous in terms of cost and ease for mass production. Though the emerging technology [19,20,21,22,23] (such as memristor, magnetic tunnel junction, carbon nanotubes, graphene and so on) based designs show attractive performance, they may not be applicable to the industry now. Therefore, we focus on introducing the CMOS based designs in this survey.

Table 1 gives a brief summary of dynamic/static entropy.

This survey paper systematically reviews and summarizes some existing works that are focused on entropy extraction from silicon-based devices of two security primitives: TRNG and PUF. The rest of this paper is organized as follows. Section 2 gives the definition of entropy. Section 3 introduces the method of harvesting entropy from TRNG, which also primarily covers the potential dangers and threats associated with TRNG. Section 4 studies the methods of obtaining entropy from different PUF models and analyzes the possible risks and attacks. The unified design of TRNG-PUF is presented in Section 5. Section 6 studies and analyzes the typical applications of TRNG and PUF as entropy sources in the field of information security. This work is summarized, and potential future research areas are suggested in Section 7.

## 2. Entropy Definition

The essence of entropy is the internal chaos of a system. The German physicist Rudolf Clausius proposed the concept of entropy in 1865 [24]. It was initially defined in thermodynamics as the rate of change of the input heat relative to the temperature in a reversible process.

(1) $$dS={\left(\frac{dQ}{T}\right)}_{reversible}$$

where *T* is the thermodynamic temperature of the substance, dQ is the heat input during the heat conduction process, dS is the essential entropy change, and the subscript “reversible” indicates a reversible process.

Around 1877, Boltzmann proposed that the entropy of a system and the number of all possible microstates satisfy the following simple relationship:

(2) $$S=k_{B}\ln\Omega$$

This formula is called the Boltzmann formula, where $k_{B}$ is the Boltzmann constant, and Ω is the total number of microstates contained in the macrostate of the system. According to this formula, entropy is a measure of the degree of distribution of microstates. In 1948, Shannon extended the concept of entropy in statistical physics to the process of information channels [1]. The entropy defined by Shannon is called Shannon entropy or information entropy, i.e.,

(3) $$H\left(X\right)=-\sum_{x\in X}p\left(x\right)\log_{2}p(x)$$

where *X* is a discrete random variable, and p(x) represents the probability of *x* in the random variable. The lower the probability of a statement being correct, the more uncertainty it has, and thus the more informative it is. Shannon entropy quantifies the mean informative.

The joint entropy of two discrete random variables, *X* and *Y*, refers to the information entropy of the element pair (x,y) of *X* and *Y*. The joint entropy is defined as follows:

(4) $$H\left(X,Y\right)=-\sum_{x\in X,y\in Y}p\left(x,y\right)\log_{2}p(x,y)$$

Conditional entropy refers to the entropy value of *X* when the random variable *X* is given by the random variable *Y*. Conditional entropy is defined as follows:

(5) $$H\left(X|Y\right)=-\sum_{x\in X}p\left(y\right)\sum_{y\in Y}p\left(x|y\right)\log_{2}p(x|y)$$

The relationship between conditional entropy, joint entropy and information entropy is

(6) H(X,Y)=H(Y∣X)+H(X)

In cryptographic system analysis, minimum entropy is the most conservative measure of the unpredictability of a set of outcomes. Formally, the minimum entropy of a random variable *X* is

(7) $$H_{min}\left(X\right)=-\log_{2}\left(max_{x\in X}p\left(X=x\right)\right)$$

In this paper, without specifically mentioning it, we use Shannon entropy.

## 3. TRNG

Generally, the device that is responsible for producing random numbers is called an RNG. An ideal RNG should generate patternless, independent, and identically distributed numbers or bit streams. There are two kinds of RNG: PRNG and TRNG. The primary distinction between them is that the output of PRNG depends on the initial seed, while TRNG harvests randomness in the uncontrollable process of entropy sources.

The evaluation of randomness for a TRNG (or the entropy source) is not easy. It is even harder than the design of TRNG itself. Basically, mainstream methods to test randomness are entropy estimate, standard statistical tests, robust estimate and attack analysis.

Shannon-entropy (Equation (3)) and minimum-entropy (Equation (7)) estimates are widely used in TRNG for estimating the nominal and worst-case entropy of the random output, respectively. Standard statistical tests, such as Crypt-X [25], ENT utility [26], TestU01 [27], Diehard [28] and National Institute of Standards and Technology (NIST) statistical test suite [29], are used to check if an input bitstream is patternless and equally distributed among “0” and “1”. Robust estimations require the under-test TRNG to work at various conditions, of which temperature and voltage sweeps are mostly adopted. The results of entropy estimates and statistical tests can reflect the robustness of the design. Furthermore, attack analysis helps with optimization with respect to attack resistance.

In this chapter, we will analyze how to build a TRNG, starting with the reviews and discussions of pseudo random and true random.

### 3.1. PRNG

The main method of establishing PRNG, or deterministic RNG (DRNG), is to build mathematical models or formulas. The seed of a PRNG is utilized to produce random bit sequences. As a result, PRNGs are unable to generate any kind of entropy or a truly random bitstream, and their output is entirely determined by their input seed. Nevertheless, in some circumstances, PRNG can increase and compress the entropy of the input seed.

**Figure 1:**
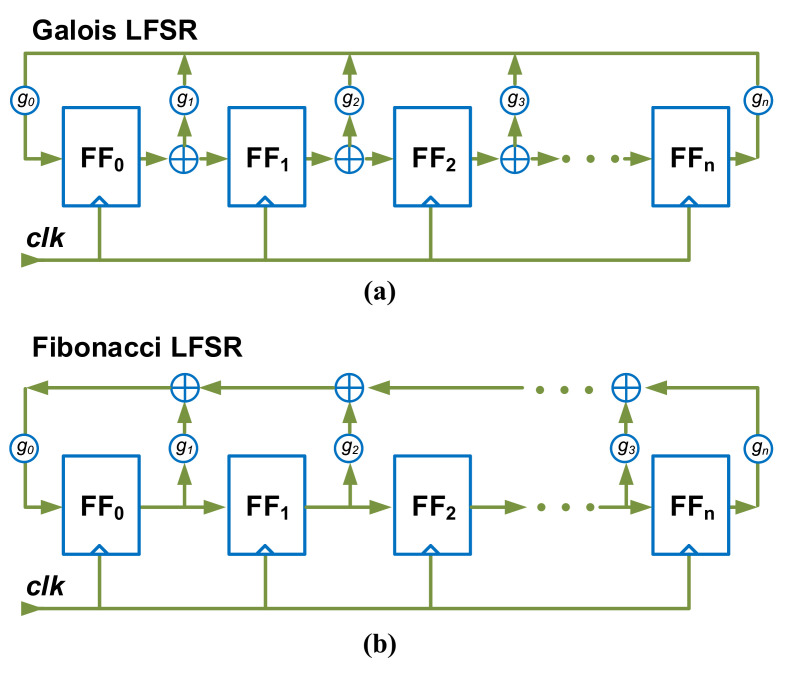
Two general types of LFSR: (**a**) Galois LFSR, (**b**) Fibonacci LFSR.

Linear PRNGs have low consumption and assure high throughput. Linear feedback shift registers (LFSRs) are a widely used class of PRNG [30,31,32,33]. As shown in Figure 1, the majority of LFSRs are comprised merely of flip-flops (FF), XOR, and ADD operations, which are compact and conducive to digital design. Linear congruential generator (LCG) is a popular algorithm for PRNG [34,35,36]. The storage-bit truncation operation, implemented in computer hardware, can realize modulo arithmetic. The following equation defines the output pseudo-random value $X_{i}$:

(8) $$X_{i+1}=\left(aX_{i}+b\right)\;\mathrm{mod}\;m$$

where $X_{i}$, *a*, *b* and *m* are the *i*-th output pseudo random value, multiplier, increment and modulus, respectively.

**Figure 2:**
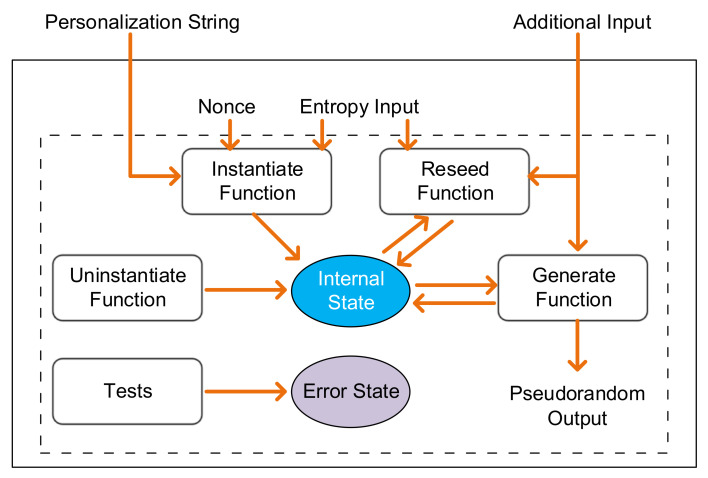
A CSPRNG mechanism in NIST Special Publication 800-90A.

Cryptography and security applications make extensive use of random numbers and random bits. The three main components of a cryptographically secure RNG (CSRNG) are an entropy source, an algorithm for accumulating and providing random bits to the consuming applications, and a way to combine the first two components appropriately for cryptographic applications. In NIST Special Publication 800-90A [37], a CSPRNG functional model is discussed in detail (shown in Figure 2), where the entropy input is provided to a CSPRNG mechanism for the seed.

The tests (evaluations) for CSPRNGs are stricter than for other PRNGs. For example, CSPRNGs should pass the next-bit test. It is proved that if an RNG passes the next-bit test, then it can pass all other polynomial–time statistical tests for randomness [38]. In addition, CSPRNG is usually implemented with a many-to-one function, i.e., the hash function, to make the guess of the reverse mapping very hard.

**Figure 3:**
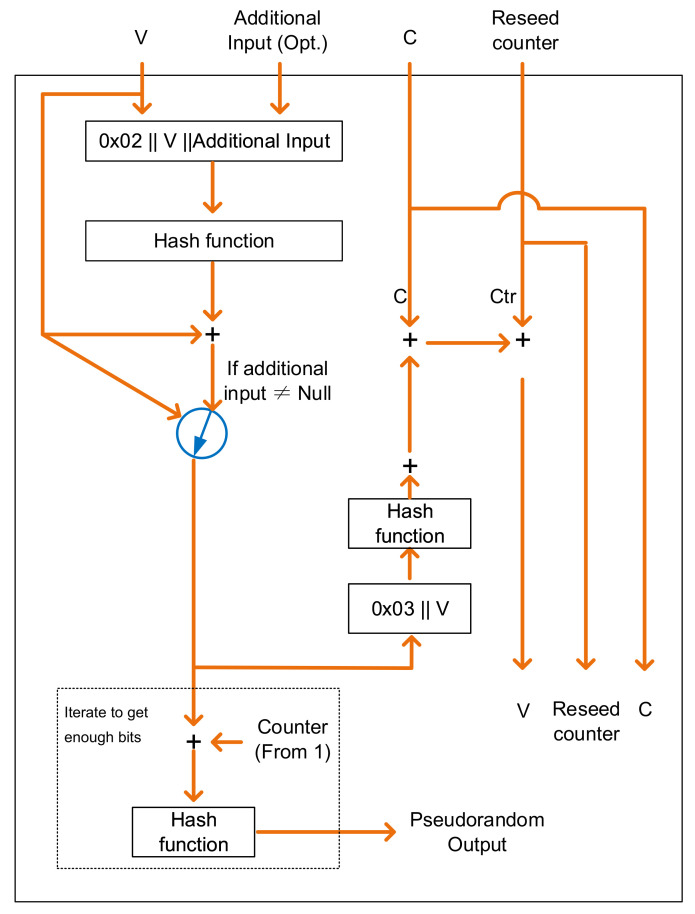
A hash based CSPRNG: Hash_DRBG.

A hash-based CSPRNG is shown in NIST Special Publication 800-90A as known as Hash_DRBG. The details of the pseudorandom bits generation function are shown in Figure 3. The hash function is used in instantiate, reseed and generate processes. V is updated whenever DRBG is called during the process, while C depends on the seed. A counter (reseed counter) records the number of requests for pseudorandom bits.

The values of V and C are the secret values on which the security of the CSPRNG depends. The security strength of such a DRNG is the security strength of the hash function for pre-image resistance.

Secure Hash Algorithm-3 (SHA3) [39] is a standard cryptographic algorithm that produces 224, 256, 384, and 512 bits of hash values by using internal bits of 5∗5∗64=1600 bits length. Earlier Secure Hash Algorithm-1 (SHA1) and Secure Hash Algorithm-2 (SHA2) used a fixed-size memory block that is 512, 1024, etc., but this is not compulsory in SHA3, and it can vary according to the requirement. The size of the message is of infinite length in SHA3, which makes it more powerful than the previous versions [40].

**Figure 4:**
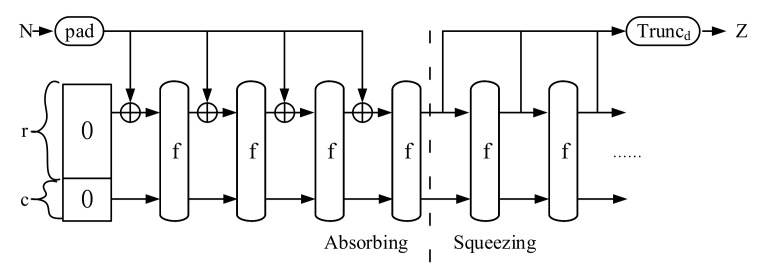
SHA3 sponge function in [39].

SHA3 uses a new Sponge Function, named Keccak, that makes it more secure as compared to earlier. The Keccak permutation, in contrast to other Merkle–Damgard-based hash algorithms, operates on a state with a fixed size of *b* bits. In the Keccak-f permutation (f[b]), the state size b can be 25, 50, 100, 200, 400, 800, 1600. Yet, Keccak f[1600] was chosen for the SHA3 standard, which can be represented as a 5×5×64 bits 3D array. The initializing, absorbing, and squeezing phases of the Keccak permutation are shown in Figure 4.

### 3.2. TRNG

In contrast to PRNG, TRNG captures randomness in entropy sources.

**Figure 5:**
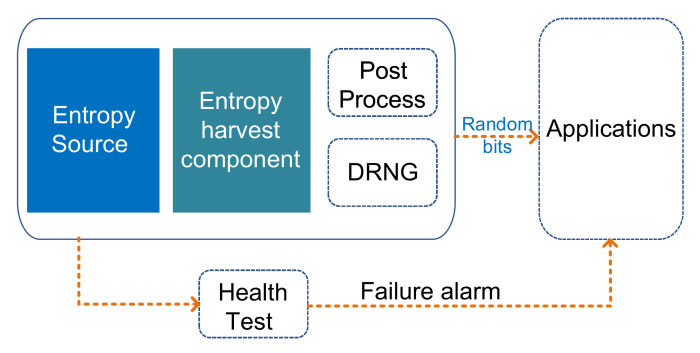
TRNG model.

A TRNG is a security primitive which produces unpredictable and random numbers. Figure 5 depicts a TRNG with a universal design. The random output of TRNG should not be predictable, even if all the design details (e.g., schematic, algorithm, timing, and operations) are well known.

Due to its non-periodicity and non-reproducibility, an ideal TRNG can guarantee the security of the information system. The entropy source is the root part of TRNG, while the entropy harvesting components and post-processing also rule the TRNG designs. The entropy source provides all the unpredictability. The goal of the entropy harvesting component is to maximize the capture of randomness. The existence of post-processing techniques that whiten the spectrum and remove bias from raw data depends on the security requirements and the caliber of the raw data.

TRNG can be divided into two categories based on the composition: (1) a pure TRNG (PTRNG) implementation without complex post-processing; and (2) a hybrid PTRNG that applies design elements from DRNGs and PTRNGs, where the additional complex mathematical post-processing can be utilized as security anchor.

#### 3.2.1. Entropy Sources of TRNG

A TRNG can also be divided by the types of entropy sources and entropy harvesting components. In this subsection, we will discuss the entropy source in the integrated circuit (IC) chips.

In integrated circuit design, noise is inevitable and undesired. However, in TRNGs, enlarging and capturing unpredictable and human-uncontrollable noise sources are the root principles. For a good entropy source model, the source itself should be white noise with a Gaussian distribution. Thus, a good entropy source is consistent with the observed properties of noise.

- Electric noise

Thermal noise, shot noise, and flicker noise (1/f noise) are the three basic types of electric noise. Thermal noise is presented in any device (such as diodes, triodes, and metal oxide semiconductor field effect transistor (MOSFET)) with resistance, caused by the random collision of electrons with thermally excited atoms that is analogous to the Brownian motion of small particles in a liquid. Thermal noise will persist unless all devices are superconducting and are approximately expressed by

(9) $$\bar{i_{thermal}^{2}}=2kTg_{m}$$

where $i^{2}$ is the current variation, *k* is Boltzmann’s constant, *T* is the temperature and $g_{m}$ is the transconductance of the device. Thermal noise is white, which is a good entropy source. The power spectral density of thermal noise drops gradually to 0 at up to a few hundred terahertz; in other words, it is easy to harvest thermal noise in gigahertz.

Shot noise is caused by unavoidable random statistical fluctuations of the electric current when charge carriers traverse a gap, which was clarified by W. Schottky. The shot noise is white noise and can be written as

(10) $$\bar{i_{shot}^{2}}=2qI_{D}\Delta f$$

where *q* is the electronic charge, $I_{D}$ is the average value of a series of random independent pulses, and Δf is the bandwidth in hertz. Shot noise increases with the bandwidth of measurement, whose standard variance is

(11) $$\sigma =\sqrt{\bar{i^{2}}}=\sqrt{2qI_{D}\Delta f}$$

The 1/f noise originates from carrier number fluctuations (CNF) [41] in MOSFETs. The dynamic charge trapping could also induce fluctuations of the carrier mobility, giving rise to the correlated mobility fluctuations (CMF) [42,43,44]. The noise voltage of 1/f noise is given by

(12) $$\bar{v_{1/f}^{2}}=\frac{K}{C_{ox}WL}\frac{1}{f}$$

where *K* is a process variable, *W* and *L* are size parameters of MOSFET, and $C_{ox}$ is the gate dielectric capacitance per unit area. 1/f noise is directly inverse to the frequency *f*. The noise current of 1/f noise decreases with the increase in frequency, while the current of thermal noise remains constant. There is a corner frequency, above which 1/f noise dominates. Below the corner frequency, thermal noise dominates. The 1/f noise may also cause a correlation of the RNG output according to Equation (12). The 1/f noise is less effective than a TRNG that works at a higher frequency.

B. Chaos

This category of entropy source is based on a deterministic chaotic system or circuit. As the random states and output are generated from the deterministic rules, it seems weird for a TRNG. However, a chaotic map is particularly sensitive to the initial conditions, so the chaotic map can be triggered by nondeterministic physical processes (for example, environmental noise or electrical noise generated) that result in long-term unpredictability [45,46,47].

Generally, a chaotic map should be driven by a clock signal, which makes the throughput adjustable, dynamically, at runtime. Chaotic maps can be classified into two categories, continuous time and discrete time, depending on whether the current state is related to the previous states. The continuous time chaotic maps are mainly established by analog devices [48]. A discrete-time chaotic map can be implemented by digital a circuit IC that can be constructed by only a few digital devices [49]. Thus, a good digital chaos-based TRNG has great potential in lightweight hardware implementations.

C. Jitter noise

The difference between the actual clock and the ideal clock in the time domain is known as jitter, and it is an important statistic for assessing the reliability of the clock signal. There are many causes of jitter, including equipment noise, power noise, external interference, load changes, and so on.

In TRNG designs, ring oscillators (ROs) are a crucial component in producing jitter. The following analysis theoretically explains the principle of TRNG design based on jitter noise. The total jitter of the RO in the strong inversion can be expressed mathematically as [50]

(13) $$\sigma_{\tau }^{2}=\frac{kT}{If_{0}}\left(\frac{2}{V_{\mathrm{DD}}-V_{th}}\left(\gamma_{N}+\gamma_{P}\right)+\frac{2}{V_{\mathrm{DD}}}\right)$$

where *k*, *T*, *I*, $f_{0}$, $\gamma_{N}$, $\gamma_{P}$, $V_{th}$ and $V_{\mathrm{DD}}$ are the Boltzmann constant, absolute temperature, saturation current, oscillation frequency, noise coefficients of negative channel metal-oxide-semiconductor (NMOS) and positive channel metal-oxide-semiconductor (PMOS) transistors, threshold voltage and power supply voltage, respectively.

The jitter variance also depends on the operating region of CMOS transistors. The subthreshold region contributes significantly higher noise currents since the diffusion currents and transconductances are relatively small in the subthreshold region. The total jitter generated by the inverters conducted in the weak inversion (subthreshold region) is given by [51]

(14) $$\sigma_{\tau_{w}}^{2}=\frac{q}{If_{0}}\left(1+e^{-V_{\mathrm{DD}}/2U_{t}}\right)$$

where *q* is the Coulomb constant, and $U_{t}$ is the thermal voltage given as (kT)/(q). To maximize entropy, some designs bias the RO to the subthreshold region, which can also significantly save energy consumption [52]. Due to their simple structure and efficiency, TRNGs using phase jitter have been extensively researched [3,4,53].

D. Metastability

**Figure 6:**
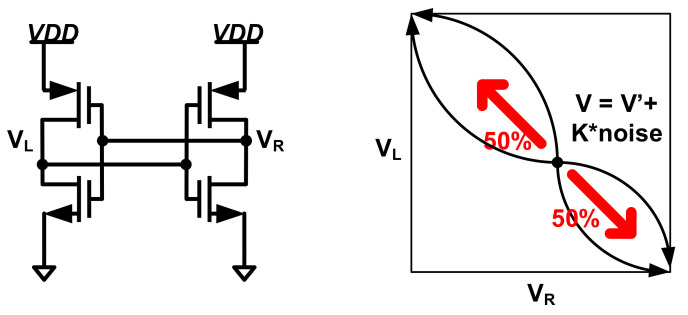
Metastability of two inverters.

The metastability is a phenomenon, an undefined or unbounded duration state, that can be attained by a circuit or a system before it is set to a more stable state. The following is an illustration of metastability for two inverters (Figure 6):

An unexpected state caused by metastability in cross-coupled inverters, latches, D-Flip-flops (DFFs), and SRAMs can be used to create a random bit stream at a high bit rate [54,55,56,57]. The major problem with this random source is that metastability suffers from process variations. Thus, post-processing units are required in most designs.

#### 3.2.2. Entropy Harvest Method/Components

In this chapter, we focus on TRNG designs that originate from solid-state devices or can be easily implemented and integrated on-chip with other circuit modules for mass production. Most silicon TRNGs are designed to harvest physical or environmental randomness using one of four entropy sources, namely, noise-based, chaos-based, jitter-based, or metastability-based TRNGs. Some representative TRNG designs are summarized in Table 2.

- Noise-Based TRNG

Noise-based TRNG utilizes electronic noise to generate random bitstreams. For this category of TRNG design, the common challenge is that the noise amplitude is always orders of magnitude smaller than the digital output of the TRNG. Thus, many of these TRNGs are equipped with operational amplifiers to boost the noise magnitude for entropy capturing [58,59], which can consume significant power and area. In addition to traditional analog-to-digital converter (ADC) methods, time-to-digital converter (TDC) methods are widely used to quantify noise in noise-based TRNGs.

Normally, the noise-based TRNGs are easier to achieve a high throughput due to the superiority in the distribution of electrical noise mentioned above.

**Figure 7:**
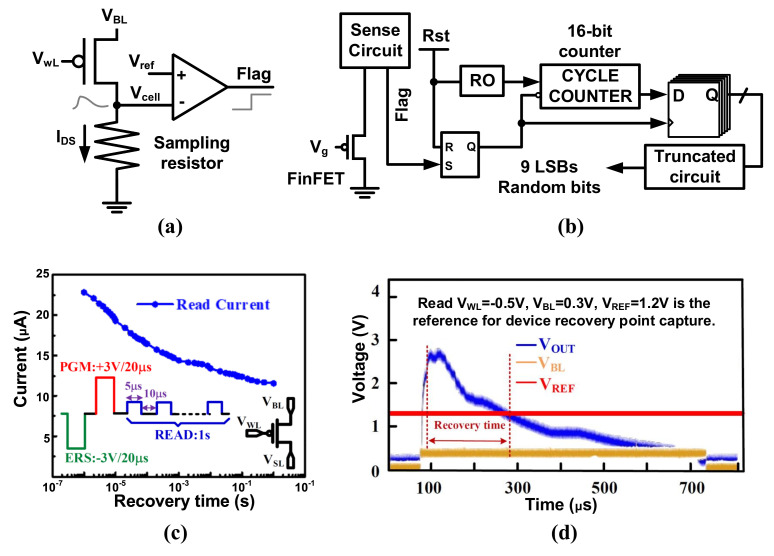
The TRNG using stochastic short-term recovery of CT-FinFET in [60]. (**a**) The sense circuit, (**b**) the entropy extraction circuit, (**c**) the erase (ERS), program (PGM) and read (READ) operations of CT-FinFET, and the work current dropping curve within 1 s after PRG, showing a short term recovery characteristic. (**d**) The sensing scheme of measuring the recovery time of CT-FinFET.

Recently, a novel TRNG design was demonstrated utilizing stochastic short-term recovery of charge-trapping fin field-effect transistor (CT-FinFET) devices [60]. The main idea to harvest the noise is to digitalize the recovery current. The sensing scheme for measuring the recovery time of CT-FinFET is shown in Figure 7a. The $I_{DS}$ is captured by a resister, and $V_{cell}$ is compared with a $V_{ref}$ to output $V_{O}$, which is the Flag signal. The short-term recovery of the CT-FinFET is repeatedly sampled using an RO-based time-to-digital count converter (TDCC) unit and then serialized into a bit stream (shown in Figure 7b), whose throughput is up to 1.5 GHz.The typical read current curve of a FinFET device is shown in Figure 7c, and the short-term recovery time can be traced by a read voltage signal given on the gate. The counter stops as the FLAG jumps from “0” to “1” when the traced voltage is smaller than $V_{REF}$ as shown in Figure 7d. Thus, the short-term recovery is converted to the 16-bit output of the TDCC. Due to the existence of noise, the recovery time is slightly different, which impacts the least significant bits (LSB) variations of the 16-bit output. In [60], it is reported that the short-term recovery exhibits a stochastic nature in the 9 lowest-order bits of the count number that pass the NIST tests without post-processing.

**Figure 8:**
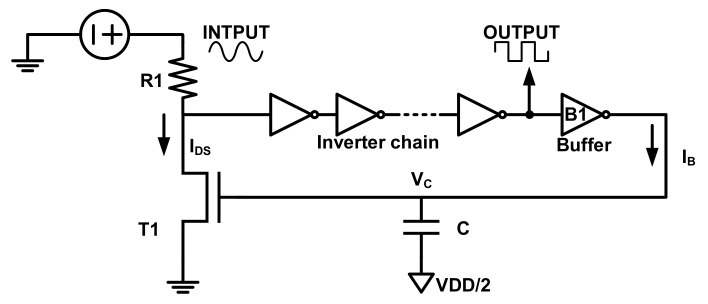
The dynamic voltage feedback tuning structure.

Another representative noise-based TRNG design is demonstrated utilizing a dynamic voltage feedback tuning (DVFT) mechanism to guarantee the feasibility and robustness of TRNG harvest randomness from the power supplier [63]. The noise generated by various power suppliers shows promise based on their measurement results. Since the Gaussian distribution of the noise implies that half of the distribution is greater than the mean and half is less than the mean, discriminating the voltages between, above and below the mean can produce random streams. As shown in Figure 8, benefiting from the DVFT design, no amplifier is used to prevent the TRNG from the exhausting area and power consumption. In an ideal case, R1 drops the power voltage to (VDD/2). For ideal inverters, (VDD/2) is the balance point to capture noise. Considering the random noise, these inverters act as amplifiers to amplify the input variations and push the output away from (VDD/2) to 0 and VDD. The addition to the TRNG circuit is the DVFT, which includes a buffer (B1), a pre-charged capacitor (C), and a transistor (T1). The buffer B1 is used to isolate the TRNG output from the feedback; the capacitor C integrates the past zeros and ones; and the transistor T1’s effective resistance varies with the voltage driven by C. When the inverter chain’s input is not near (VDD/2), assuming $V_{G}S$ of T1 is increased, $I_{d}s$ is also increased. As $I_{d}s$ increases, so does the voltage drop across R1, lowering the voltage at p1, the inverter chain’s input to (VDD/2). When voltage at the input of the inverter chain decreases, the DVFT circuit tunes the voltage by an opposite feedback process, which results in the self-adjusting TRNG mechanism. Under the DVFT mechanism, the inverter chain captures the variations of power supplies and outputs random bits.

B. Chaos-based TRNG

**Figure 9:**
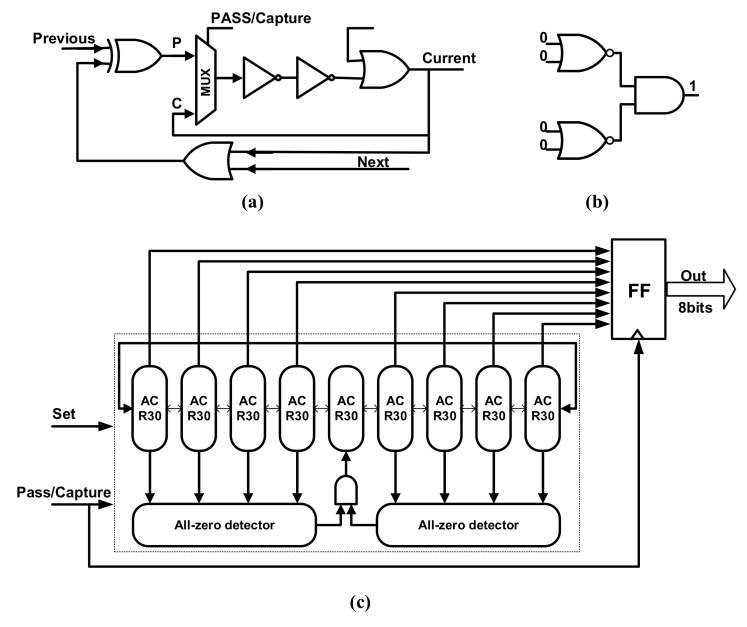
(**a**) The asynchronous circuit of RC30 (ARC30). (**b**) All-zero detector. (**c**) The TRNG design based on ARC30.

Luo et al. [49] present a novel TRNG design method based on a chaotic cellular automata 30 (CA30) topology. The state evolution of the CA30 scheme is chaotic [64], and the authors have implemented an asynchronous circuit realization of CA30 (ACR30) to harvest random behaviors. For the whole design shown in Figure 9, nine ACR30s are employed to set up the self-timed ring, and eight of them are used to generate true random number sequences. Eight DFFs are clocked by the Pass/Capture signal to sample the random bits. Since the ACR30 has a “stable” state that can lock the state of the oscillation chain, two detectors are added to pull out of the “stable” state of the ACR30-based ring structure. The footprint of this schematic is approximately equivalent to 75 NAND gates, which is a very lightweight design.

Due to the purely digital nature of the chaotic TRNG design, the authors can realize this TRNG structure in FPGA devices, which consume only 53 LUTs and 22 DFFs in total and achieve a power efficiency of 8.2 pJ/byte. The practical measurements of the TRNG chip achieve a power efficiency of 0.63 pJ/bit at a clock speed of 250 MHz, which takes the auxiliary circuits and IO pins into consideration, and the achieved throughput is 1 Gb/s. The harvested random numbers passed all the tests of NIST SP800–22 with a high passing rate and passed all the IID test cases with a minimum entropy of 7.07026 (8-bits) in NIST Test Suite SP800–90B.

C. Jitter-based TRNG

**Figure 10:**
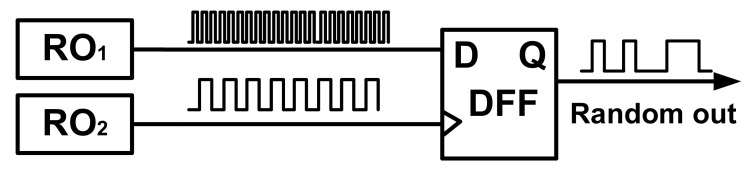
RO-based TRNG.

Figure 10 shows a conventional design of oscillator-based TRNGs, whose randomness is derived from the instability of oscillating signals caused by the noise (i.e., jitter) in the circuit. The entropy source consists of two oscillators, for example, RO. The slow RO samples the output (oscillating signal) of the fast RO in a sampling unit, such as a D flip-flop.

Another method to extract jitter noise is to utilize TDC (time-to-digital converter) as the entropy harvester [61,65,66]. As it is discussed in Section 3.2.2 A, TDC is utilized to measure or capture slight time intervals between two signal edges. When the entropy source is jitter, TDC can trace signal edges, and convert the pulse width to binary data. The jitter is contained in oscillation edges, and the output of the TDC has captured entropy, which is generally shown in the LSBs of the TDC output.

**Figure 11:**
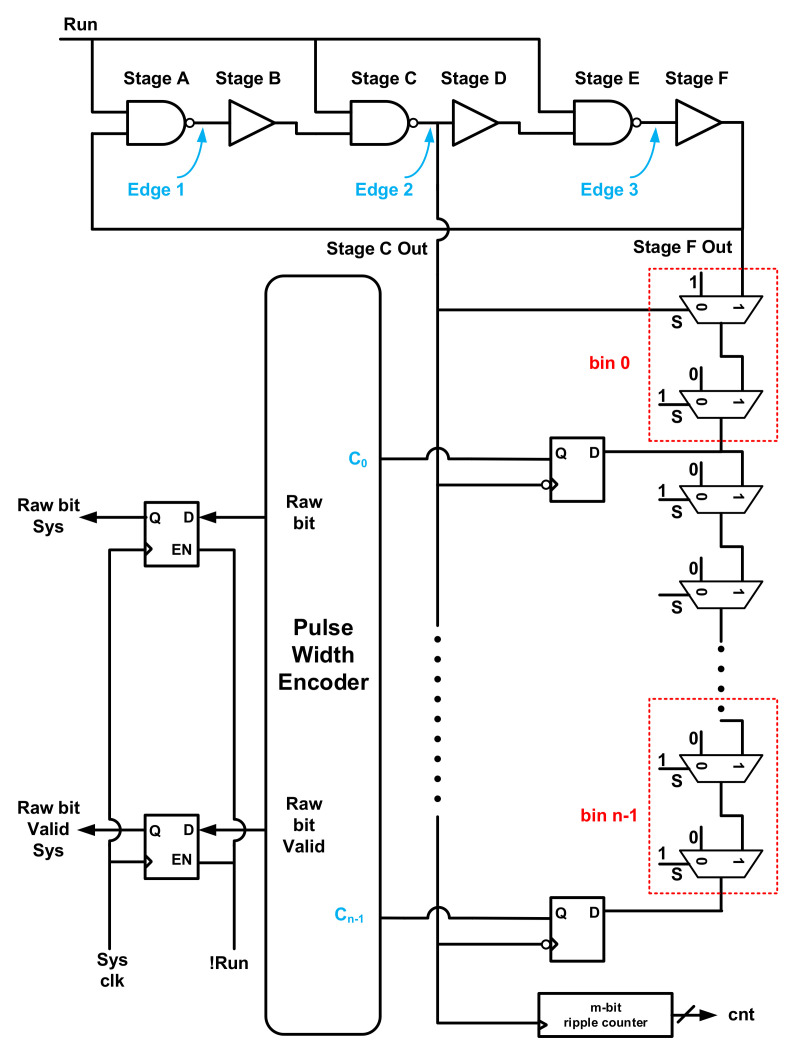
Three-edge RO-based true random number generator with time-to-digital conversion TRNG.

In [61], TDC effectively acquires jitters accumulated independently by each edge, as shown in Figure 11. Once the run signal is valid, the six stages will have an identical mean period and inevitably collide due to noise influence. The “Stage F OUT” is connected to a delay chain, where the time of the adjacent oscillating edges is digitized. When the run signal fails, edge 1 will trigger the corresponding DFFs to sample the state of the delay line, where the DFF output $C_{0}-C_{n-1}$ is encoded into the TRNG raw bits. Through post-processing based on the binary linear codes [67], the proposed TRNG occupies 33 slices and achieves a throughput of 12.5 Mbps with post-processing of Golay code on a Xilinx Zynq-7000 FPGA, and the generated bit streams pass the NIST SP 800-90B test and the AIS-31 test.

D. Metastability-based TRNG

**Figure 12:**
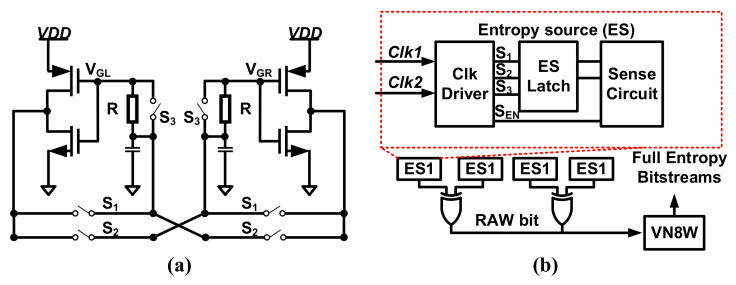
(**a**) Latched cell and (**b**) TRNG core presented in [62].

Recently, a latch-based TRNG that harvests the metastable region’s enhanced random noise with 8-bit von Neumann post-processing was presented in [62] as shown in Figure 12.

To enhance the noise, the equalization phase of the two inverters in the latch is divided into a low-resistance (LR) phase and a high-resistance (HR) phase by turning S3 on and off, respectively. In the LR phase, the gate and drain voltages in each inverter are quickly equalized. In the HR phase, the RC delay time is added, and a damped oscillation is introduced. Thus, small noise is amplified into large-amplitude noise with a random phase. An additional sense circuit is utilized to read $V_{GL}$ and $V_{GR}$ differences, as shown in Figure 12b.

The entropy harvesting component consists of four entropy sources (ESs) and 4-bit XOR circuits. To remove residual bias and correlations, 8-bit von Neumann post-processing with waiting (VN8W) is used. While VN8W has a larger area overhead, it brings higher throughput and higher energy efficiency for the TRNG core. The randomness of the TRNG output is verified by the NIST SP 800-22 and NIST SP 800-90B tests. The fabricated chips achieve a power efficiency of 0.186 pJ/bit at 0.3 V, consume a core area of 661 $\mu m^{2}$, and a total area of 5561 $\mu m^{2}$ including VN8W. Furthermore, to verify the power noise injection attack tolerance, one chip with two TRNGs is measured under a supply noise frequency range of 0.1–59.335 MHz with a 1.1× growth step. According to the test results, the proposed TRNG has shown robustness against power noise injection attacks, whose output passes the NIST SP 800-22 and NIST SP 800-90B tests as well.

### 3.3. Post Processing

The randomness of TRNGs may be weakened due to the PVT variations [68]. Post-processing is used to correct the statistically flawed output raw stream. It not only masks the defects of output bits, but also increases throughput in some applications. The widely used simple correctors are XOR [69,70], LFSR [71], von Neumann corrector (VNC) [72] and so on. Post-processing can also be as complicated as resilient function [73] and hash function [37].

The XOR correctors gather the TRNG raw bits from several entropy sources and XOR them altogether to produce a 1-bit output. XOR correctors require that the number of entropy sources is greater than 1, but it reduces the throughput. The more entropy sources there are, the higher the entropy will become in the output bitstreams.

The LFSR correctors are seeded by the raw bits and expand the random output by a pseudo-random mechanism. The risk of this kind of post processing is that if the entropy of the input bits is low, the pseudorandom will predominate in the output bits, crashing the true random and decreasing entropy.

VNC is an ideal method to reduce bias. It compares pairs of bits and outputs “1”, “0”, or “null” ([1,0] = 0; [0,1] = 1; other = null). However, when this method is used to deal with highly biased data, the output rate is greatly reduced.

These correctors can be implemented online or offline, but the security of these algorithms is hardly guaranteed when an attacker targets the entropy source. NIST special publication 800-90A actually recommends employing one of the proven post-processing methods based on cryptographically secure primitives, e.g., block ciphers or cryptographic hash functions with a health test mechanism, to make TRNGs cryptographically useful. Yet, the obvious drawback of cryptographically secure post-processing is that the extra power overhead and area is unbearable for lightweight applications.

### 3.4. Risks and Attacks

Attack analysis can also be used to evaluate the TRNG randomness, which is strongly related to system security. It is a common practice in applied cryptography to check the security of all building elements independently. For this reason, evaluating the robustness of the generator and all its parts is of great interest [74].

**Figure 13:**
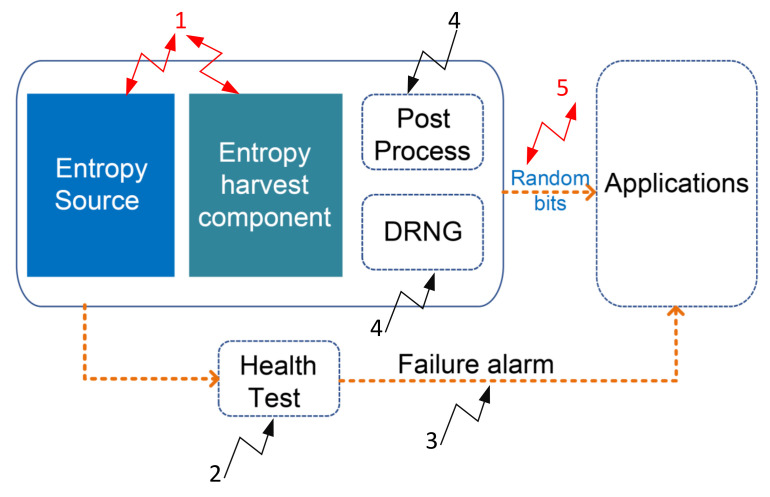
Passive (1, 5) and active (1, 2, 3, 4) attacks on a general TRNG structure.

It is reported that attacks on the RNGs are possible, both passively and actively, such as side-channel attacks (SCA) [74], fault injection attacks [75,76,77] and machine learning attacks [78]. With the rapidly advancing machine learning algorithms, new challenges are coming soon. A general structure of possible attacks is shown in Figure 13. Active attacks tend to modify the behavior of the generator to control its output. In contrast, passive attacks collect some information about the generator to predict future values with a non-negligible probability or to easily tune an upcoming active attack.

SCA obtains privacy from side-channel information, including timing, power, electromagnetic and other physical signals, when edge devices interact with the external environment. Bayon et al. [74] showed the vulnerability of RO-based TRNG to the EM active noninvasive attack. To retrieve the information on the RO-TRNG embedded in the device, they used a so-called differential frequency analysis. Since ROs are more sensitive to the environmental conditions than those of the system clock, RO frequency contributions to the power spectral density (PSD) can be discerned by performing differential frequency analysis. Benefiting from this, the authors successfully retrieved the frequency and location of the oscillating ROs. Having retrieved the information on the location of the RO-TRNG and its working frequencies, it is able to properly tune the active electromagnetic attack. The test results in [74] show that if under attack, the two ROs are synchronized and operated at the same frequency, and the TRNG would not pass the complete test suite.

**Figure 14:**
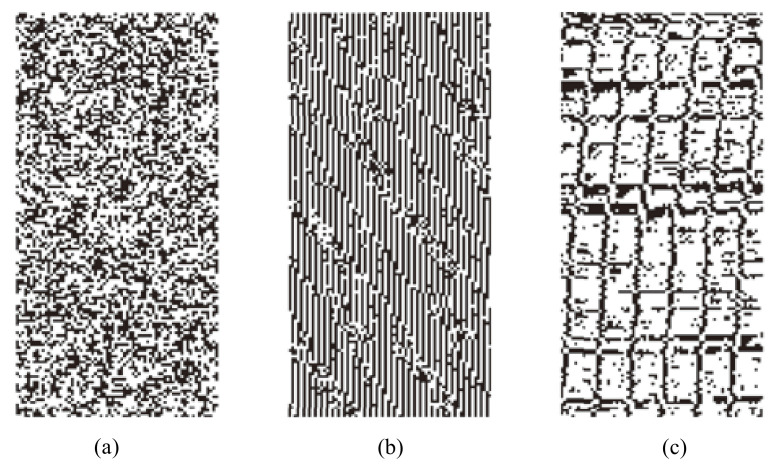
Frequency injection attack results on a TRNG which was implemented in [77]. (**a**) no injection, (**b**) 1.822880 MHz injection and (**c**) 1.929629 MHz injection.

A fault attack introduces deliberate faults into the computation of the cryptographic function and exploits the faulty results to extract information about the secret key. Environmental elements that can be used for injecting faults are varied, such as high temperature, ultralow temperature, strong electromagnetic, and strong light environment [75]. For example, most oscillator-based TRNGs are vulnerable to frequency injection attacks [76,77]. The oscillation phase of free-running ROs can be locked by injecting frequency into the power. As a result, the white entropy source (jitter) is destroyed. Figure 14 shows the attack result on a TRNG in automated teller machines. The dark dots represent “0”, and the white dots represent “1” in the TRNG output.

**Figure 15:**
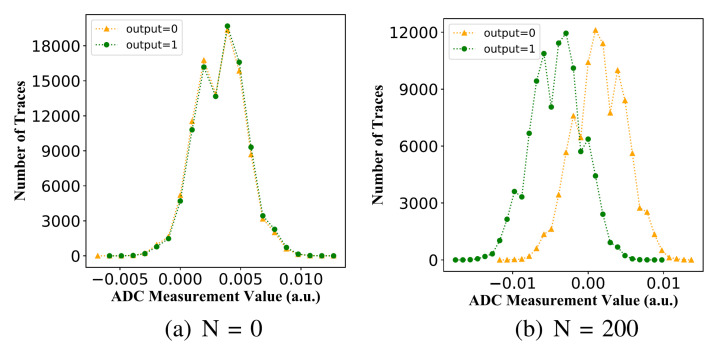
Power consumption of MURO-TRNG core [78]. (**a**) The origin design, (**b**) 200 extra flip-flops are added. Note that N is the number of added FFs.

Recently, a deep-learning-based SCA was developed to attack a TRNG, which was implemented on FPGA [78]. The original MURO-TRNG is presented in [73]. To implement the SCA, a bitstream modification process is used to add extra flip-flops (FFs) into the original MURO-TRNG core, where the distribution of power consumption overlaps can be distinguished (shown in Figure 15). If 200 extra flip-flops are added to the bitstream of the device under attack, an accuracy of close to 100% can be achieved, using the proposed bitstream modification process. Strictly speaking, the deep learning attack in [78] has not broken the core since the extra bitstream modification process is necessary. However, machine learning attacks remain a potential threat, and they have demonstrated some possible trends for successfully attacking a TRNG with the insertion of a hardware Trojan into the original design or chip.

To protect the design from attack over a larger range of noise, decoupling capacitors can be placed close to the power rail or around the ROs in the layout [79]. In terms of countermeasures to attacks and environmental variations, structure optimization [80] or a reliable online health test mechanism [3,81] is necessary.

## 4. PUF

PUF derives entropy from the physical properties of the integrated circuit (IC). Each chip varies, owing to manufacturing unpredictability. PUFs extract the static entropy from manufacturing process fluctuations as opposed to TRNGs. Once the chip is constructed, the manufacturing process differences are coagulated and alter very little during the course of the chip’s lifetime. As a result, we can refer to this type of entropy as static entropy.

The primary function of PUF is to serve as a source for identification and authentication. Every device should have a distinctive label in order to ensure genuine authentication. To explain the usage, two concepts are proposed, intra-PUF variation and inter-PUF variation, which are calculated by the Hamming distance (HD). Intra-PUF variation and inter-PUF variation are also called inter-die HD and intra-die HD, respectively. Intra-die PUF variation is defined as the number of bits in a PUF response that vary when an identical challenge is repeatedly queried on a given PUF device in a given environment, while inter-die PUF variation is defined as the number of bits in a PUF response that vary between different devices for a set of shared challenges. For the application of secure authentication, intra-PUF variation should be low so that the PUF can be verified. On the other hand, inter-PUF variation should be high (ideally 50% on average) so that two separate PUFs have maximally decorrelated responses.

**Figure 16:**
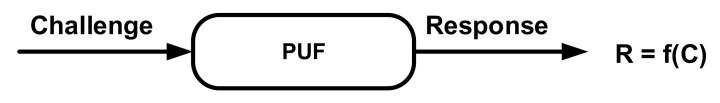
PUF model.

Basically, a PUF generates a sequence (response) of the unique signature by input initial states (challenge), so-called challenge–response pairs (CRPs). Each PUF can be represented as a black box, R=f(C), as illustrated in Figure 16, where the f() is secret.

The PUF circuits tend to be robust and small in size, which makes them well suited for radio-frequency identifiers (RFIDs), smart cards, and other small and low-cost internet-of-things (IoT) devices [82].

Based on the number of CRPs, PUF can be divided into two categories: weak PUF and strong PUF. Strong PUFs are typically used for authentication, while weak PUFs are used for key storage. Explicitly stated, weak PUFs have the following properties:

- A small number of CRPs (grows linearly with area or unit component).
- Response is reproducible and stable to a certain challenge, and robust to the environment.
- Response is random and unpredictable, which only depends on the process variations of IC.
- Even if the f() is leaked, the mapping of response and challenge pairs cannot be rebuilt in another device or chip.

The weak PUFs can generate only one or a limited number of CRPs [83].

In contrast, the requirements for strong PUFs are as follows:

- The number of CRPs must be very large, which makes it impossible for the opponent to enumerate all CRPs in a fixed time. The CRP space grows exponentially with the area.
- CRPs should be stable enough to be effective against ambient conditions and multiple readings.
- Open access mode: any entity with access to a strong PUF can apply multiple stimuli and can read out the corresponding responses. There are no incentives and no response to PUFs that are protected, controlled, or restricted access.
- Security: neither the attacker nor the manufacturer of the PUF can correctly predict the response to a randomly chosen stimulus. This conclusion holds, even if the above two parties can access strong PUF for a considerable period of time, even with proper physical measurements.

Strong PUFs provide enough CRPs for authentication without the need for any additional cryptographic hardware. However, any internal functionality of significant PUF leaking is prohibited. On the other hand, a robust PUF may be sufficiently mined for CRPs to allow for the training of machine learning models that can accurately predict CRPs.

In terms of randomness, PUF was evaluated similarly to TRNG. However, there are differences in the robustness tests. Uniqueness and reliability are the most important indicators in PUF evaluation. Uniqueness measures the ability to distinguish two identically designed PUF instances. It can be measured by calculating the inter-die HD:

(15) $$\text{inter-die}\;\mathrm{HD}=\frac{2}{m(m-1)}\sum_{u=1}^{m-1}\sum_{v=u+1}^{m}\frac{\mathrm{HD}(R_{u},R_{v})}{n}\times 100\%$$

where $R_{u}$ and $R_{v}$ are the n-bit response of two different chips, *u* and *v*, to the same challenge *C*. The inter-die HD is usually measured for the appropriate number of chips in a nominal environment for hundreds or thousands of challenges.

The reliability of a PUF can be measured by the intra-die HD of its responses when the same challenge is applied to the same PUF instance. Some designs also use the bit error rate (BER) to evaluate the reliability. The intra-die HD is expressed as follows:

(16) $$\text{intra-die}\;\mathrm{HD}=\mathrm{BER}=\frac{1}{k}\sum_{j=1}^{k}\frac{\mathrm{HD}(R_{i},R_{i,j})}{n}\times 100\%$$

where the $R_{i}$ is an n-bit response to an input challenge C produced by a PUF chip *i* under the nominal operating condition, and the same set of challenges are then applied *k* times to obtain the response $R_{i,j}$ for j=1,2,…,k.

### 4.1. PUF Models

In this section, the architectures and models of typical silicon-based PUFs are discussed. Weak PUFs and strong PUFs are discussed separately due to their different mechanisms. Some representative PUF designs are summarized in Table 3.

#### 4.1.1. Weak PUF

- Oscillator PUF

**Figure 17:**
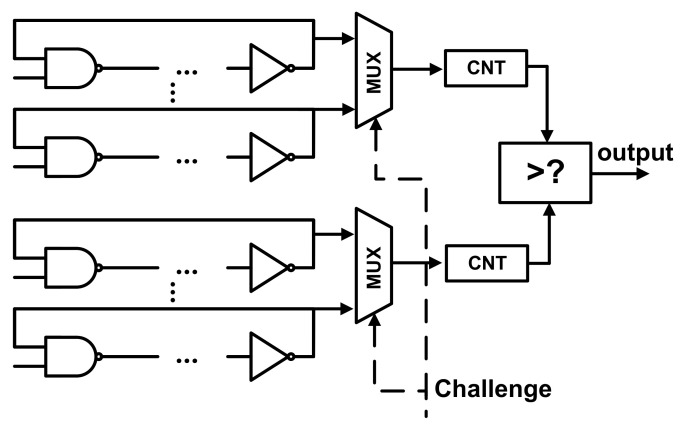
RO-based PUF.

Oscillator PUF [10,11,89] contains *N* identically designed oscillators, and transforms the frequencies of free oscillating oscillators to binary data by counters. The frequencies are compared to generate response bits. The entropy source of this category of PUF is unpredictable mismatch delay variations in every single delay stage, where the oscillators’ frequency relays on. RO PUF is a widely implemented PUF, which is shown in Figure 17. If there are *N* ROs, then the number of possible pairings is N(N−1)/2. However, the number of CRPs is limited due to correlations. Thus, RO PUF is a weak PUF.

Note that the responses of RO PUF are measured by counters. The RO PUF is susceptible to the same set of environmental variations and noise sources. As a result, some research has concentrated on error correction or PVT resistance in applications [84,90].

B. SRAM PUF

**Figure 18:**
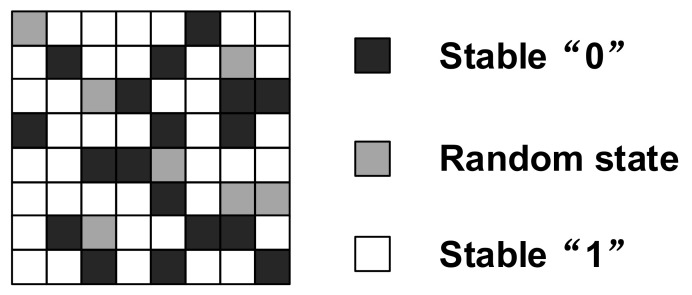
SRAM PUF block.

**Figure 19:**
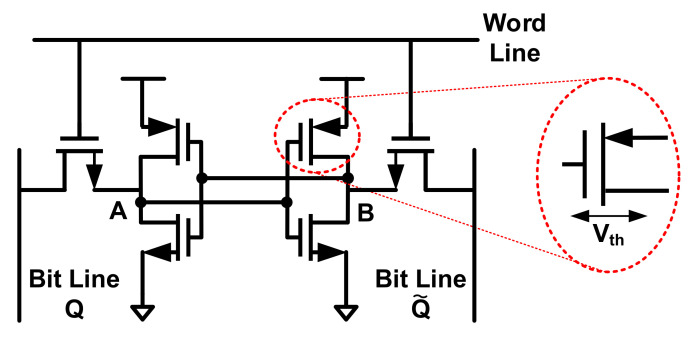
SRAM cell. The $V_{th}$ mismatch results in the SRAM powering up in either a logic “0” (A = 0, B = 1) or logic “1” (A = 1, B = 0).

A popular weak PUF structure exploits the positive feedback loop in an SRAM [12,91,92]. The basic block is shown in Figure 18. The challenge to an SRAM PUF is a memory address, while the corresponding PUF response is the content of the uninitialized memory cells at this address. A basic SRAM cell is shown in Figure 19. The SRAM cell has two stable states, stored as “1” or “0”, and the feedback mechanism will force the cell to fall into a state when the cell is powered up, protecting the storage data from noise. When no write operations are made, the ideal SRAM cell has an equal opportunity to be pushed into “1” or “0”. Yet in actual designs, the transistors’ threshold is slightly different, which results in a certain state of the feedback loop due to the process variations. Thus, the SRAM PUF is reached. However, a small number of SRAM cells are unstable and show stochastic states at every power-up. Since the feedback loop in SRAM is controlled only by process variations, if the two feedback loops show enough similarity, the noises result in an output bit flip. To sum up, SRAM PUF is applicable to either generating reliable secure keys (reliability and uniqueness requirement) or providing random entropy to the device (randomness requirement) [12]. Thus, most SRAM-based PUFs are implemented with proper corrections.

In [86], to improve the stability of responses in SRAM PUF, PMOS is added as an SRAM power switch to guarantee an ns-level $T_{ramp}$ (the time it takes for VDDs to increase from zero to a supply voltage VDD) of SRAM cell, which can decrease the power-up time and drop the probability of bit flips. When the chip is powered on, the SRAM power switch is turned on by default. Then, the power on SRAM values is labeled as $R_{A}$. When VDD is stable, they turn off the SRAM power switch and reboot the SRAM after a time of $T_{sleep}$. With an ns-level $T_{ramp}$, the VDD of SRAM rapidly increases from zero, and the power-up value is represented as $R_{P}$. The $R_{P}$ achieves a worst-case BER of 5.35% at 1.5 V core VDD, 85 °C, and a $T_{ramp}$ < 1 ns with these actions. Under the same conditions, the $R_{A}$ has a worst-case BER of 13.4%, which is significantly higher.

#### 4.1.2. Strong PUF

- Arbiter PUF

**Figure 20:**
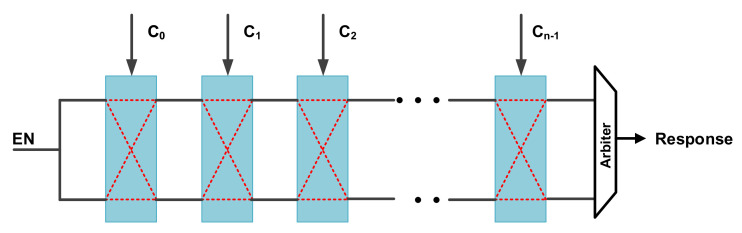
The basic APUF.

An arbiter PUF (APUF) is a delay-based strong PUF that has a race condition between two symmetrical digital paths. Each delay stage contains two multiplexors that are controlled by challenges ($C_{0}∼C_{n-1}$) shown in Figure 20.

When it begins to work, after a trigger signal is activated, the trigger signal is driven on two paths that are determined by a pre-input challenge and end in an arbiter, who determines which of the two paths is faster to generate the binary response that fits the black-box model (R=f(C)). APUFs can be efficiently implemented in application-specific integrated circuits (ASICs) and FPGAs [82,87].

B. Other representative strong PUF

Generally, the PUF, whose response is dependent on all entropy source units, is easy to guarantee a large number of CRPs and constitute a strong PUF [93,94,95]. In the case of APUF, the 1-bit response is defined by all the delay units. The selection of delay units at each stage is determined by the challenges ($C_{0}∼C_{n-1}$). Thus, the race result at the final arbiter is the integral over the delay time of all challenge-affected paths. Because all delay units affect the 1-bit response at the same time, the CRP space of an N-stage APUF is 2n.

**Figure 21:**
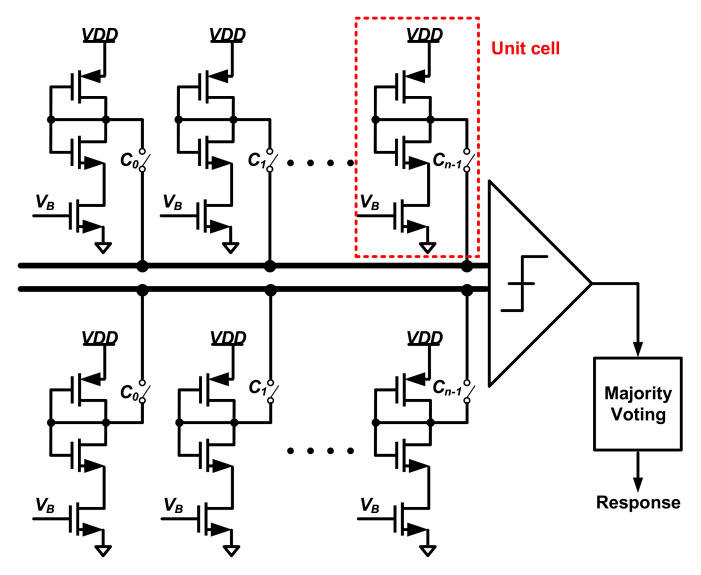
The subthreshold voltage-divider-based strong PUF.

The essence of signal racing is the comparison of voltages at a given moment. In terms of voltage comparison, Venkatesh et al. present a subthreshold voltage-divider-based strong PUF in [88], which is shown in Figure 21. The arrangement of the voltage divider is similar to an APUF. The challenge inputs, $C_{0}$ through $C_{n-1}$, determine which of the N unit PUF cells in both arrays are connected to the differential inputs of a comparator. A 1-bit response is generated by comparing the drain voltage of two symmetrical voltage dividers. $V_{1}$ and $V_{2}$ are controlled by challenges. It is a strong PUF with a $2^{n}$ CRP space.

### 4.2. Risks and Attacks

As PUFs are proposed for authentication and key generation, reproducibility and robustness should be ensured. PUFs, however, are vulnerable to some attacks.

#### 4.2.1. Working Conditions

**Figure 22:**
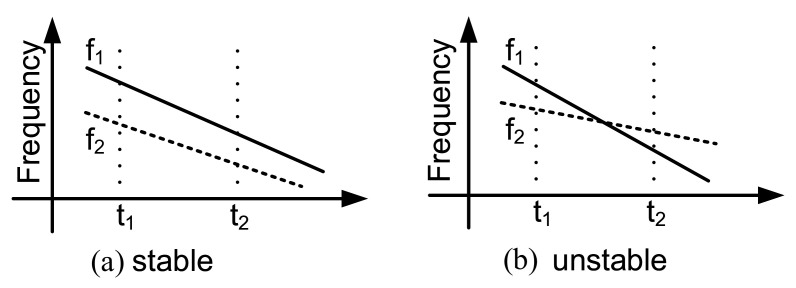
If the oscillation frequencies of the two selected ROs have a large difference in temperature sensitivity, a response bit flip can occur.

PUFs are sensitive to working conditions, especially temperature and voltage. The BER gradually increases as temperature and voltage shift away from the reference state. For example, the RO-based PUFs compare the frequency of challenge selected ROs to generate a response bit. The frequency of challenge selected ROs will vary due to temperature sweep, which may result in an error bit (shown as Figure 22).

To be resistant to temperature variations, ROs are designed to be robust to temperature in [84]. The regular inverters of the ROs are replaced by current-starved (CS) inverters to improve the reliability of the PUF. By leveraging on the extra gate biasing of the CS inverter to control its drain current, an optimal bias is determined to account for the counteracting effects of temperature and supply voltage on RO frequency. Even in the worst case, the reliability is still above 94%.

#### 4.2.2. Silicon Aging

The quality of PUFs suffers from several noticeable degradations and hard faults due to silicon aging [96]. These degradations and faults cannot be rectified, and they make a PUF chip unreliable to use. In order to explore the real effect of aging and eliminate the interference of dynamic entropy, an extremely mass of CRPs should be collected multiple times, and their BER should be measured. For example, in [97], each SRAM PUF chip was read out around 11 million times, and 16 devices were tested. Through long-time miscellaneous measurements, the reliability of SRAM PUF worsens within a limited boundary due to the aging effect, whose BER increases by about 0.74% each month over a 2-year period. The proportion of stable PUF bits decreased from 85.9% to 83.7%. Mohd et al. also demonstrated that the reliability of TCO-PUF [98] and arbiter-PUF degrades by about 4.5% and 2.41%, respectively, after 10 years, while RO-PUFs and SRAM-PUFs degrade by about 12.76% in 10 years and 7% in 4.5 years, respectively [99].

**Figure 23:**
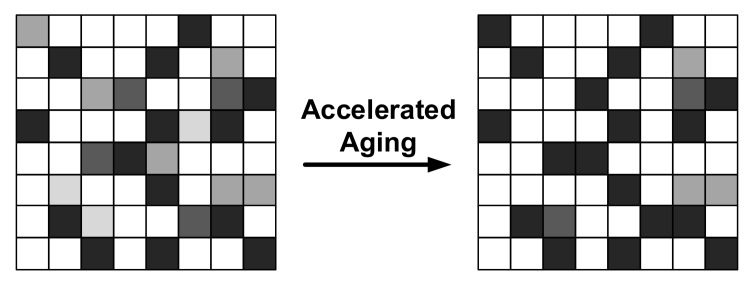
An example of SRAM PUF that improves reliability against aging by pre-aging.

Additionally, the reliability of some PUFs can be enhanced by accelerating aging. For instance, the reliability of SRAM PUF can be improved by increasing the magnitude of the difference in the threshold voltages of the two PMOS devices in the cross-coupled inverters [100,101]. The effect is shown in the Figure 23.

#### 4.2.3. Modeling Attack

Weak PUFs have a limited number of CRPs, which limits their use mainly to random key generation, and their challenge–response interfaces are usually obfuscated to prevent direct access to exhaustively rebuilding the CRPs for playback or spoofing attack. Strong PUFs have an exponential number of CRPs which cannot be exhaustively measured within a reasonable time, which makes them well suited for IoT device authentication [102]. Due to the large CRP space, high response reproducibility to the same challenge under varying operational conditions after device manufacturing is harder to achieve, and modeling attack by machine learner becomes feasible. It may be hard for a strong PUF design to resist a machine learning attack merely by sophisticated design alone without considering the nature of data science.

For a simple 64-bit APUF, the ML algorithm can predict the response with a high accuracy of 95% when the training data only contain 640 CRPs, while 18,050 CRPs are needed to achieve a high accuracy of 99.9%. Take the BER of PUF itself into consideration. It is demonstrated that the arbiter PUF can be learned efficiently [103].

Numerous variations of APUF have been proposed to resist ML attacks. Unfortunately, most of these designs failed in the new attacks [104]. Moreover, Ahmad et al. presented a new machine learning procedure for attacking the XOR arbiter PUF. In their design, smaller training datasets are needed and achieve a higher efficiency for large XOR PUFs [105]. In other words, it is possible to attack a strong PUF successfully that relies merely on static obfuscation.

To thwart machine learning attacks, designers must deal with how deep learning networks work. There are several solutions to be considered:

- Do not give it enough data for training. Very sophisticated deep learning is very powerful against very complex design modeling. However, it requires a lot of data to obtain an accurate prediction. For authentication, if the mechanism generates a response only once or a few times, it is acceptable, even if the response is very slow in many applications. For attackers, it may end up taking years to collect the training data. “SHIC PUF” deliberately and significantly slows down the response generation time from the input of a challenge to lengthen the time required to collect the response by the attacker [106].
- Contaminate the data accessible by the attackers. There are some design ideas that poison the response data collected by the attackers, and the legitimate user knows how to differentiate the true and fake responses [107]. In [108], by adding some extra models, such as PRNG and a fake PUF, an active deception protocol was guaranteed to prevent ML attacks.
- Make use of dynamic characteristics/parameters. If the CRP space can change with dynamic characteristics or parameters (time, e.g.), then even if the attacker can build a successful model from previously collected CRPs, it will not be useful for breaking the same PUF after its CRPs have been refreshed.

#### 4.2.4. Side Channel Attack on PUFs

SCAs are powerful non-invasive attacks. Mahmoud et al. proposed the first side-channel boosted ML attack on XOR-based PUFs [109]. When the PUF is initialized such that the inputs to the XOR gate are all zeros, and the total power trace is measured. Because no two paths are exactly the same, a unique glitch in the power trace can be measured when an APUF response switches from “0” to “1”. Thus, APUFs with a response of “1” can be extracted.

SCAs are combined with ML methods to achieve a reduction in the size of training data and attack time. In [110], an optimized attack is discussed, which includes weight vector estimation based on linear programming and generating new CRPs using the cutting-plane method. By implementing the attack with SCA in [109], the simulation results show an extensive reduction in attack complexity compared with previously proposed ML-based attacks. It achieves an average reduction of 66% in attack time.

## 5. TRNG-PUF United Design

Since the entropy sources of traditional TRNGs and PUFs are different, TRNGs and PUFs are individually designed in different modules or chips. However, it is expected to have both hardware security primitives (TRNG and PUF) in a single chip to strengthen the security of security applications [111,112]. Furthermore, the constrained resources in IoT devices have led to the unified designs of secure cores, such as ADC-based TRNG/PUF and SRAM-based TRNG/PUF. There are a few PUF and TRNG unified designs reported in the literature [5,113,114,115] which is a new trend in entropy harvest designs.

**Figure 24:**
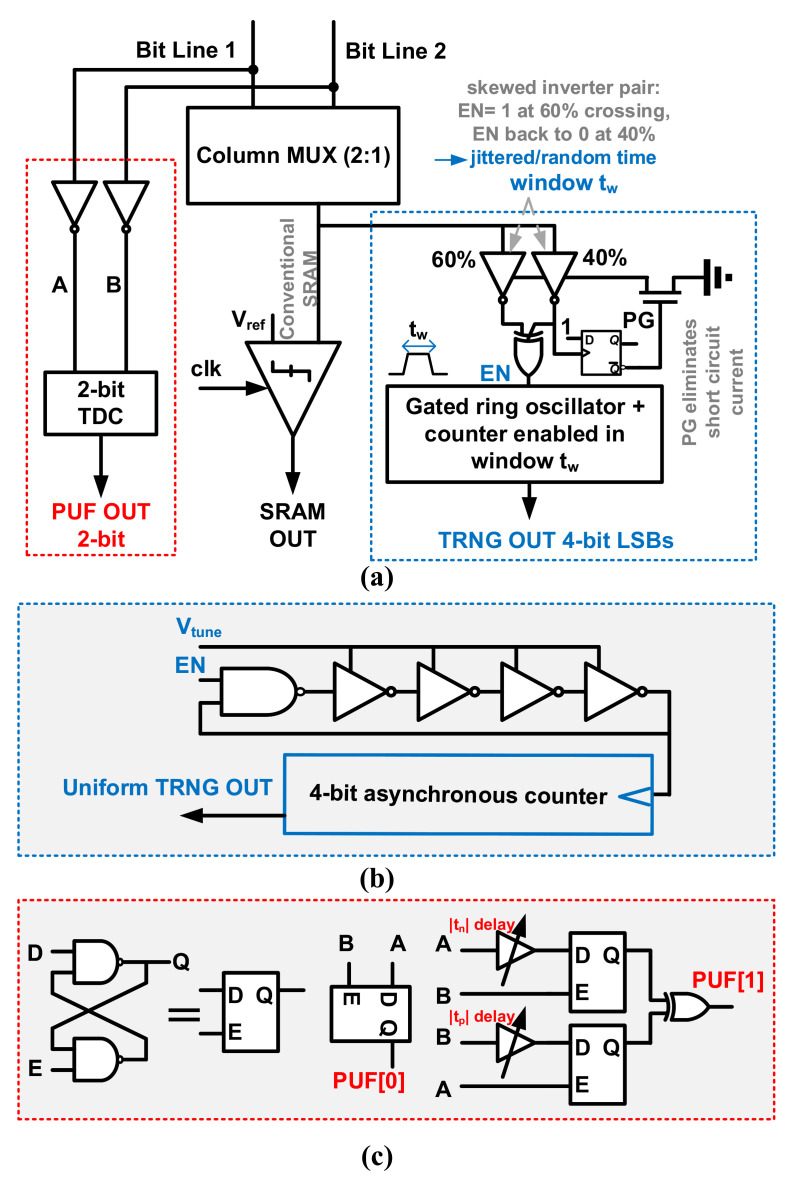
In-memory unified TRNG and multi-Bit PUF. (**a**) The core circuit design of unified TRNG and PUF, (**b**) random number generation by RO-based TDC, and (**c**) 2-bit TDC for PUF generation.

SRAMs are widely used as PUFs and TRNGs in commercial chips due to their ubiquitous availability, but the quality of existing SRAM-based TRNGs is limited due to the limited amount of entropy [12,116]. In [113], Sachin et al. proposed an SRAM with unified TRNG and multibit PUF for complete in-memory dynamic and static entropy generation method as shown in Figure 24. In this design, two categories of TDCs are connected to the bitline and trace the bit changes. The random behavior of the SRAM bitline discharge rate, which is caused by electronical noises, is used to produce TRNG. A 4-bit RO-based TDC is utilized as the entropy extractor. When PUF bits are asked, two bitlines are racing at bit change speed. The static entropy is harvested by the 2-bit TDC, which is similar to an arbiter.

In [113], the TRNG digital output is generated by digitizing the jittered bitline discharge time due to leakage via the RO-based TDC. When the SRAM data are changed from “0” to “1”, the EN is driven by a skewed inverter pair during the bitline voltage crosses 60% to 40% of VDD. The time of the jittered bitline voltage crossing the pulse window is converted to the RO-based TDC output, where the 4-bit LSBs are used as random number bits. The power source $V_{tune}$ is reconfigurable, which adjusts the frequency of RO to maintain the average count at the intended target within a threshold. As for PUF, the multi-bit static entropy per PUF bitcell is obtained by digitizing the bitline discharge time difference. Suppose the discharge times of bitline0 and bitline1 are $t_{0}$ and $t_{1}$, respectively. PUF bit0 is generated by a direct comparison between $t_{0}-t_{1}$ with 0. PUF bit1 is generated by comparing $t_{0}-t_{1}$ with a $\left(t_{p}/t_{n}\right)$ delay.

**Figure 25:**
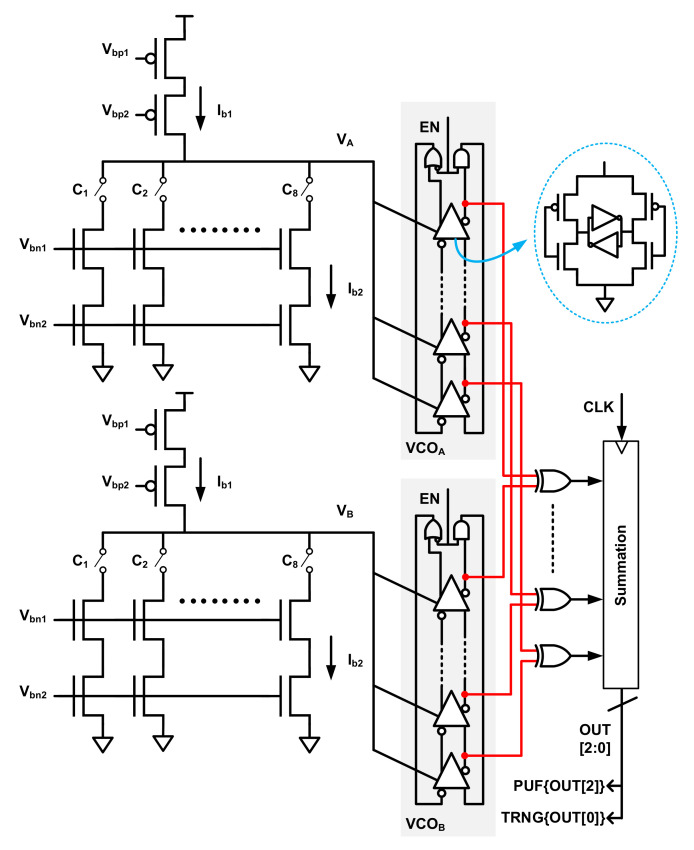
Unified analog PUF and TRNG based on current-steering DAC and VCO.

In [5], the PUF and TRNG are implemented by analog current-steering DAC and voltage-controlled-oscillator (VCO) devices, shown in Figure 25.

Two DACs are initialized symmetrically to provide the working current of two identical VCOs. Each DAC output is quantized by a ring VCO. An XOR-based subtractor extracts the difference between the quantized phase outputs of the two VCOs, which is the key method to harvesting both dynamic and static entropy. The deterministic and periodic phase difference of the DAC-controlled VCOs is the static entropy source, while the electronic noises and jitter are extracted as the dynamic entropy source. If there were no mismatches between two symmetrically set DACs, both VCOs would run at the same frequency, and the quantized output would be zero. The frequency of VCO can be written as $f_{vco}=k_{vco}*v_{\mathrm{in}}$, where the $v_{in}$ is output voltage of DAC and $k_{vco}$ is VCO’s tuning gain. Due to the mismatch in the current sources, the sampled phase difference between the two VCOs is

(17) $$\begin{array}{cc} \phi [n]= & \mathrm{mod}(2\pi k_{\mathrm{vco}}\left(1+\Delta k_{\mathrm{vco}}\right)v_{\mathrm{in}}\left(1+\Delta v_{\mathrm{in}}\right)nT_{s}\\ & -2\pi k_{\mathrm{vco}}v_{\mathrm{in}}nT_{s},2\pi )\\ \approx & \mathrm{mod}(2\pi f_{\mathrm{vco}}nT_{s}\left(\Delta k_{\mathrm{vco}}+\Delta v_{\mathrm{in}}\right),2\pi ) \end{array}$$

where $T_{s}$ is the sampling period, $k_{\mathrm{vco}}$ is the fractional random mismatch in VCO gain, and $k_{\mathrm{in}}$ is the fractional random mismatch in DAC output voltage. ϕ[n] is a deterministic, repeatable and periodic signal which depends on the mismatch between the DACs, which is the static entropy source. Thus, the static entropy module is established to create a weak PUF by sampling ϕ[n], whose most significant bit (MSB) is used as the PUF bit. In the presence of noise and jitter, the LSBs of the summation in the subtractor vary dynamically with time, where the dynamic entropy can be harvested. By running the VCO for a long time, long PUF and TRNG bitstreams can be generated. Yet, the length of PUF is limited to avoid temporal correlation.

A review of silicon-based TRNG/PUF designs is shown in Table 4. Among all of these designs [5,113,114,115], the unified implementations of TRNG and PUF demonstrated to reduce the integration effort, cost, and area.

## 6. Applications

As the promising hardware security, the research interest in PUF and TRNG has been increasing year by year, triggering a wave of research upsurge in the emerging field of IoT. TRNGs are essential in many applications, such as communication systems, statistical sampling, computer simulation, and cryptography. The unpredictable and aperiodic output of TRNG enables it to provide continuous random numbers for application scenarios, such as gambling, long-term key generation, secure seeding of hybrid random number generators, random number generation against side-channel attacks and replay attacks, etc. As an emerging lightweight hardware security primitive, PUF has the advantages of low power consumption, fast response registration, and low measurement cost, and shows excellent potential in encryption key generation and device identification and authentication applications.

### 6.1. The Applications of Random Numbers in Cryptography

The main uses of random numbers in cryptography are generating nonce, salt, initiation vector, and key (symmetric key or asymmetric key) [117].

#### 6.1.1. Nonce Generation

The nonce is the abbreviation of the number once. As the name implies, a random number that can only be used once in cryptography is called a nonce. That is, once the random number is applied, it becomes invalid and cannot be used again. A nonce can be generated whether it is a PRNG or a TRNG, and its primary function is to prevent replay attacks. In the authentication protocol or data encryption transmission system, the nonce will be used as seed data and a seed vector to participate in identification or data validity judgment.

**Figure 26:**
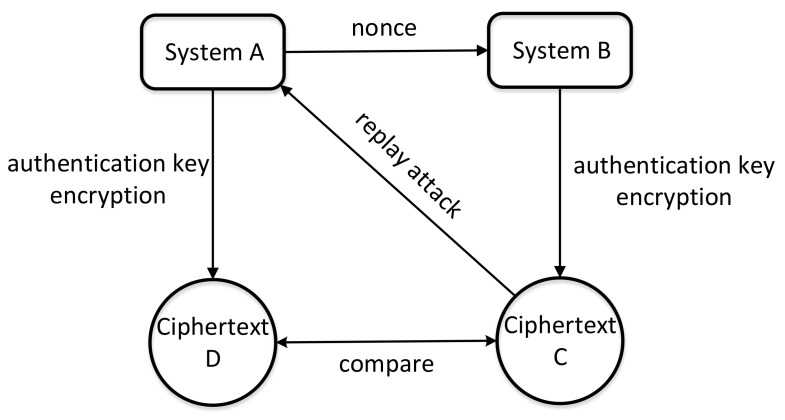
Noce-based symmetric identity authentication system and its potential replay attack model.

As shown in Figure 26, in the identity authentication system based on the symmetric algorithm, after system A generates the nonce as the authentication initiator, it sends the nonce to the unknown identity system B. Then, system B uses the authentication key to encrypt the nonce and returns the obtained ciphertext C to system A. Finally, system A uses the same authentication key to encrypt the nonce to generate ciphertext D and judges whether the identity of system B is legal by comparing the values of C and D. The role of the nonce is critical; if the value of the nonce is fixed, it means that the ciphertext C is unchanged. In this way, the attacker can intercept the authentication ciphertext C by monitoring. Every time system A initiates authentication, the attacker returns the fixed ciphertext C to system A to forge the real identity. This method is called the replay attack [118].

In a data encryption transmission system, such as a recharge system, some data play a significant role, such as account recharge information. If the nonce remains unchanged or changes regularly, the generated recharge instruction will not alter or change periodically, then the attacker can replay it through the line and can forge the recharge information to complete the illegal recharge operation on the account. Xu et al. [119] introduced a system that can recover IoT devices in a short period of time. This architecture uses TRNG as an entropy source to generate an attacker’s unpredictable nonce, thus resisting replay attacks and enhancing the security of the system.

#### 6.1.2. Salt Generation

**Figure 27:**
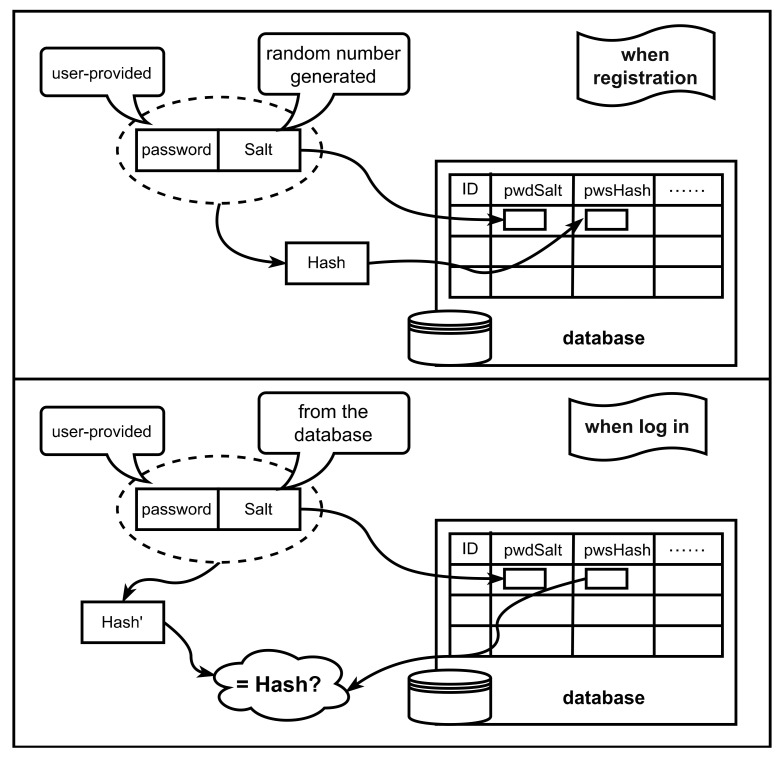
Salt + hash prevents brute force cracking to protect data security.

In cryptography, salts are random data that are used as an additional input to a one-way function that hashes data, passwords or passphrases. Salts are used to safeguard passwords in storage. Historically, a password was stored in plaintext on a system, but over time, additional safeguards were developed to protect a user’s password against being read from the system. Salt is one of those methods. Figure 27 shows the process of adding salt hash to save the password. When the user registers, the user needs to provide the password (and other user information), and then the system uses the random number to generate a salt value for the user. The system connects the salt value and the user’s password together, hashes the connected value to obtain the hash value, and then puts the hash value and the salt value into the database separately. When the user logs in, the user provides the user’s name and password, and the system finds the corresponding hash value and salt value through the user’s name. Similarly, the system connects the salt value with the password provided by the user, hashes the connected value to obtain the hash’, and confirms whether the password is correct by comparing whether the hash and hash’ are equal.

Salts defend against a pre-computed hash attack, e.g., rainbow tables [120]. Since salts do not have to be memorized by humans, they can make the size of the hash table required for a successful attack prohibitively large without placing a burden on the users. In this way, cracking becomes prohibitively expensive for hackers using rainbow table attacks, and similarly, brute-force cracking becomes unlikely. If you need to achieve a higher level of security, you can use a CPU-consuming hash algorithm to combat brute force cracking, such as password-based key derivation function 2 (PBKDF2). Ali et al. [121] discussed encryption and decryption of the dam data using the AES algorithm with derived keys via the PBKDF2 and RNG sequences generator and slave key for salting protection. They propose a derived key based on the AES algorithm plus 256 bits. The encryption result is combined with the dynamic random salt value generated by the RNG sequence generator and protected by the slave key, thus improving the security of the management system.

#### 6.1.3. Initialization Vector Generation

**Figure 28:**
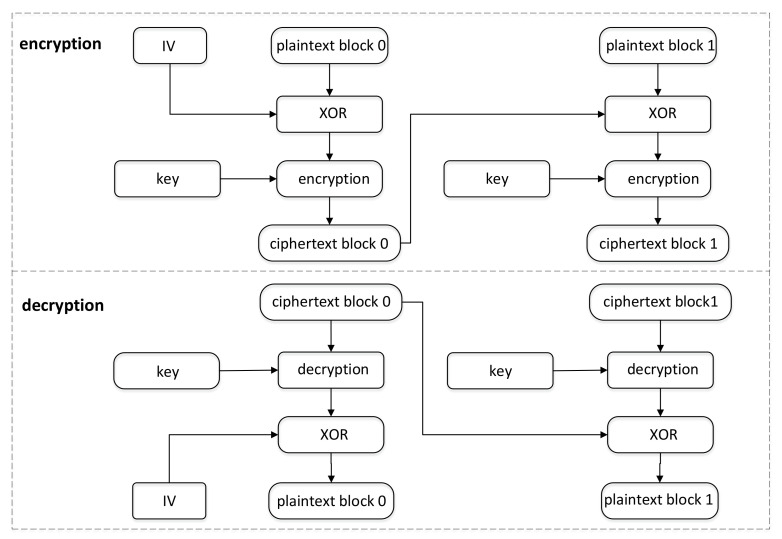
The encryption and decryption process of the first plaintext XOR with IV in CBC mode.

The random number generation initialization vector (IV) is mainly used in the cipher book chaining (CBC), cipher feed back (CFB), and output feed back (OFB) modes of block ciphers [122,123]. Taking CBC as an example, the advantage of this mode is that the plaintext information structure is well hidden, and there are nested associations between all levels of packet data. As shown in Figure 28, the first plaintext data block of this mode needs to be XORed with the IV, which can be a fixed value or random value. However, most developers or protocol specifications choose to use random IV because under the premise that the plaintext is fixed, the random IV will make the first plaintext data block a random variable, thus making each ciphertext generated by subsequent operations randomly changing. If a random IV is generated before each encryption, for the same data, the ciphertext of each encryption changes randomly. In this way, the difficulty and cost of cracking are significantly increased, which dramatically increases the strength of data security.

Al Zain et al. [124] proposed a block-based cipher scheme that uses a two-dimensional discretized chaos standard map (CSM) for encryption in three operating modes: electronic code book (ECB), OFB, and CBC. In the proposed 2D discrete CSM with OFB and CBC, the IV is used as the master key, where the IV is randomly generated to resist various types of brute force attacks. The proposed 2D discrete CSM with OFB and CBC is compared with the 2D discrete CSM with ECB. The results show that the 2D discrete CSM based on OFB and CBC has higher security than ECB from a cryptographic point of view.

#### 6.1.4. Dynamic Key Generation

In the symmetric encryption algorithm system, the one-time valid key has a critical application, called the dynamic random key, in some applications. A new key is agreed upon before each interaction between the two parties, and then the key is used for channel encryption and other processing. The significance of the dynamic random key is that the keys used by both parties change every time they communicate, and the cracker can only crack one of the historical keys but cannot break the entire system. Therefore, the cracking cost can be increased, and the security risk can be reduced. At the same time, the dynamic random key can effectively prevent line replay attacks because the key changes every time, so the same ciphertext cannot be restored to the same plaintext, and vice versa. This means that the key generation needs to be random. Take a counter as an example: if the dynamic key is regular, then as long as one key is cracked, other keys can be restored after analysis. Therefore, the randomness of the key must be guaranteed to be truly secure.

**Figure 29:**
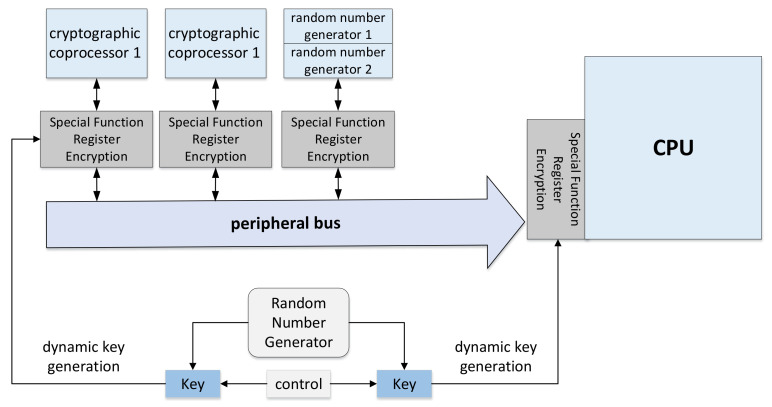
Random numbers generate dynamic key for dynamic encryption of peripheral bus.

Nowadays, more and more designers use a security chip with TRNG to generate random numbers and use the random numbers directly as keys or as a seed for generating keys. In [125], the true random number generator-pseudo random number generator (TRNG-PRNG) module is used to generate keys randomly. Therefore, in the process of encryption and decryption, the key value is difficult to be used by unauthenticated users (i.e., malicious attackers), thus improving the robustness of the architecture against malicious attackers. Figure 29 is a typical random number generator application inside a safety controller. The random number is used to generate a dynamic key to dynamically encrypt the data bus and peripheral registers so that the encrypted data transmission is realized between the CPU and the peripheral, and there is no plaintext in the whole process. Therefore, high-quality random numbers play an essential role in information security systems. If the randomness of random numbers is not secure enough, the entire system is very likely to be broken by attackers.

### 6.2. Low-Cost Authentication

There are two main applications of PUFs: low-cost authentication and secure key generation. Strong PUFs are typically used for authentication, while weak PUFs are used for key storage. This subsection introduces the application of PUF in low-cost authentication, and the application of PUF in secure key generation will be introduced in the next subsection.

#### 6.2.1. PUF-Based Authentication Protocol

**Figure 30:**
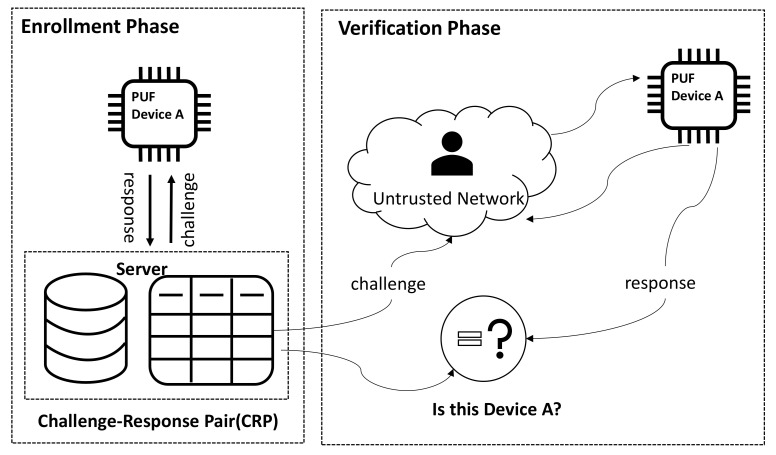
PUF-based authentication [16].

Authentication is the process between a user and a verifier who uses corroborative evidence to confirm the identity of the user [126]. Since PUFs do not require secure non-volatile memory, anti-tamper circuits, or additional support for cryptographic acceleration hardware, PUF-based solutions require less area, power, and masking layers than traditional secure authentication methods. The simplest form of a PUF-based authentication protocol proceeds in two phases: registration and authentication, as shown in Figure 30. During the registration (which happens in a secure facility), when the trusted party has a real PUF device A, a small subset of possible challenges is randomly selected and applied to the PUF to generate a corresponding set of responses. The CRP for each token is recorded by the server in a secure database for future authentication. The amount of CRP stored per token can be relatively small since the large CRP space for strong PUFs and the secrecy of the selected subset make it difficult for an adversary to construct a clone to impersonate the token. During the verification phase, the server side selects a challenge that was previously recorded but never used for the authentication operation, and obtains a PUF response from chip A. If the response matches (i.e., is close enough) to a previously recorded response, the PUF device is real.

There are a few recent research works aiming at developing PUF-based authentication protocols for the IoT. Chaterjee et al. [127] proposed a private PUF-based anonymous authentication protocol named 3PAA. The protocol allows users to anonymously authenticate the application provider (AP) *k* times through a trusted party without revealing the CRP. This makes the system more resilient to PUF modeling attacks. However, the protocol only allows the application provider to verify the authenticity of the user, but the user cannot verify that he/she is communicating with a legitimate application provider rather than an attacker. Therefore, once the AP is compromised, the security of this protocol is broken. Lounis et al. [128] proposed a novel lightweight T2T mutual authentication protocol (T2T-MAP) based on PUFs. Similar to other PUF-based authentication protocols, T2T-MAP also consists of an enrollment phase and a verification phase, but it is worth noting that in addition to performing authentication, T2T-MAP also allows the establishment of a symmetric cryptographic encryption between two transaction keys. The ability of T2T-MAP to prevent CRP leakage and resist attacks, such as machine learning and node sabotage, is stronger. In addition, the protocol also features fast authentication, reasonable communication overhead, and low energy consumption, thus achieving the characteristics of being retractable, lightweight, fast, and efficient.

#### 6.2.2. Privacy Preserving Mutual Authentication

Traditional PUF-based authentication protocol schemes risk exposing secret IDs to machine learning-based side-channel attacks that can successfully clone PUFs by analyzing thousands of challenge-response behaviors [87,129,130]. In addition, this scheme requires additional infrastructure, and the IoT remote needs to verify server credentials before outputting a response using PUF. Furthermore, each authentication routine in traditional PUF-based protocols uses a new set of challenges, which causes the channel response log to be gradually exhausted, and eventually, the product can only be retired early or recalled for new registration.

**Figure 31:**
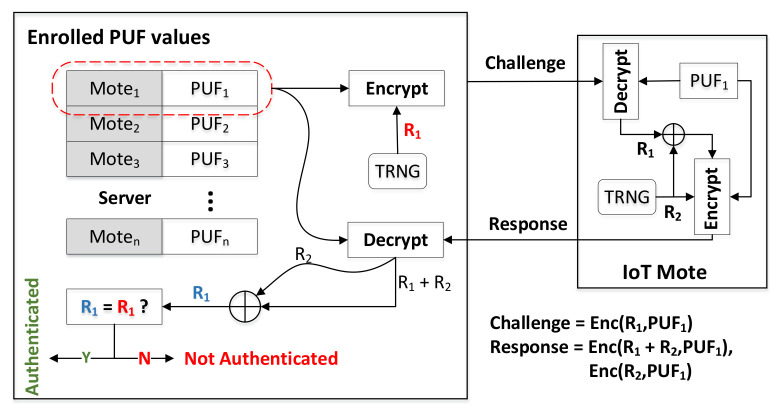
PPMA protocol.

Privacy-preserving mutual authentication (PPMA) is a recently proposed scheme. It is an authentication protocol based on the TRNG/PUF architecture. During the authentication process, the nonce generated by TRNG masks the PUF response, confusing the information exchange and replacing the traditional PUF-based authentication protocol [131]. PPMA allows the reuse of challenge–response pairs, while significantly reducing the likelihood of secret leakage. In this scheme, the server encrypts the PUF challenge with a random value $R_{1}$ generated by the TRNG before sending it, as shown in Figure 31. Subsequently, in the following authentication stage, the IoT mote (short for remote, a mote is a wireless transceiver that also acts as a remote sensor) decrypts the challenge and creates a new random value $R_{1}+R_{2}$ by combining the decrypted $R_{1}$ and its own locally generated random number $R_{2}$. After this, the IoT mote encrypts the PUF response with $R_{2}$ and $R_{1}+R_{2}$, respectively, before sending it back to the server over the insecure channel. The server uses this pair of encrypted responses to obtain a random value $R_{1}$. If the $R_{1}$ obtained from the decrypted response is equal to the random value generated by the original TRNG, then the authentication process is completed. Compared to traditional schemes where the attacker has access to the initial challenge–response value, PPMA only has access to the encrypted version, significantly reducing the scope of side-channel attacks. In addition, using a pair of random tweaks generated by IoT edge devices and servers improves resilience to replay attacks. This symmetrical use of random value $R_{1}$ and $R_{2}$ on both sides of the server and IoT mote makes mutual verification possible.

### 6.3. Secure Key Generation

**Figure 32:**
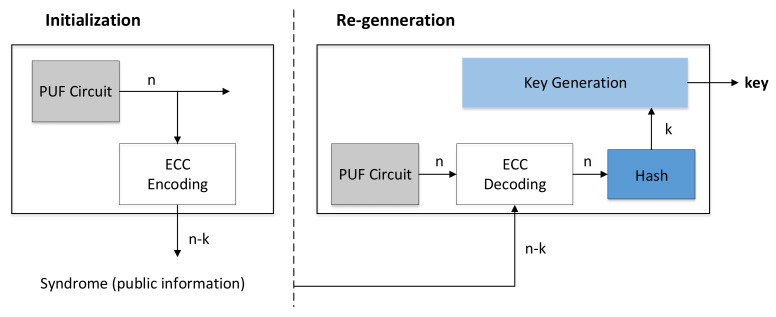
Secure key generation with PUF [10].

IoT devices with limited resources provide a challenging environment for establishing privacy and security protection mechanisms. While various cryptographic algorithms can be devised to address the above challenges, all these measures ultimately rely on securely maintained keys. Because of its limited challenge–response space, weak PUF architectures are frequently used to generate encryption keys and create passwords in communications and digital signatures to protect systems [10]. However, due to the effects of noise and altering environmental conditions, even on the same IC with the same challenge, there is no guarantee that the output of each evaluation will be the same [132]. In addition, in contrast to cryptographic primitives, such as RSA, which use keys to satisfy certain mathematical properties, the PUF output is randomly determined by manufacturing variables. Therefore, the output of the PUF is not appropriate as an encryption key directly. PUF generates a key that can be used for encryption operations, which consists of two parts: initialization and regeneration. The entire process is shown in Figure 32. Firstly, the error correcting code (ECC), consisting of initialization and regeneration, ensures that the PUF continues to generate stable output, even under significant environmental changes such as voltage and temperature fluctuations. Second, the key generation process converts the PUF output into an encryption key. For encryption operations that use a randomly selected number as the key, the output of the ECC can simply be hashed to the desired length and used as the encryption key. The hashed PUF output can be used as symmetric key for algorithms such as AES. In the initialization step, the PUF circuit generates an output, and the error correction syndrome of this output is calculated (e.g., the BCH code can be used to calculate the syndrome), which is the information that allows correction of bit-flips in the regenerated PUF output. To reproduce the same PUF output, the PUF circuit first generates the output. If there is a saved bit vector, it is used to select the pair. The PUF then uses the syndrome from the initialization step to correct for changes in the circuit output. In this way, the PUF can reproduce the output of the initialization step.

The PUF with key generation capability can be tightly integrated with a processor, enabling a physically secure processor [133]. Due to various model-building attacks [134,135], it is now recognized that it is difficult to provide security guarantees for simple challenge–response-based lightweight authentication protocols built on strong PUFs. One way to thwart modeling attacks is to limit the number of exposed CRPs by limiting the number of authentication rounds. The price of this is that PUFs must be destroyed once a predetermined number of authentication rounds is reached [136]. A recent study of strong PUF-based authentication mechanisms concluded that PUF-based secure authentication mechanisms are best constructed from PUF-derived keys [137]. In [138], a PUF-based mutual authentication protocol was proposed, which uses PUF-generated keys to authenticate IoT devices while avoiding key storage using dynamic keys.

In addition, PUF can also provide password keys for security authentication protocols based on cryptographic algorithms to authenticate IoT devices. Miguel et al. [139] described a novel anti-counterfeiting approach for IoT devices, using the unique characteristics of memory chips to derive a cryptographic secret combined with a blockchain for trusted and reliable verification of device identities. They proposed using an SRAM-based PUF to generate cryptographic keys that are employed in a zero-knowledge proof to authenticate an IoT device. In this way, even low-cost devices can sign messages by using PUF-derived keys, thereby preventing their communication with the blockchain, which makes the proposal applicable to any device with limited resources connected to the blockchain [140].

## 7. Conclusions

TRNGs and PUFs are the fundamental primitives to harvest entropy. In this paper, we highlight the importance of TRNGs and PUFs in information security systems because modern cryptography highly depends on randomness extraction.

TRNGs using electric circuits have shown wide prospects due to being carried out on compact electronic chips and thus are worth further investigation.We examine several techniques for collecting electrical noise acting as trustworthy entropy sources, including classical noise amplifiers, oscillators, metastability, and chaos. Oscillator- and metastability-based TRNGs are more portable and easier to implement, while chaos is the most suitable technique for fast random number generators. Furthermore, on-chip auto-calibration and entropy assurance are gradually showing their importance to guaranteeing the high entropy of TRNG.

PUF circuits can produce distinct, confidential information for each circuit. Two categories of PUFs, strong PUF and weak PUF, are clarified based on the CRP space. Strong PUF provides enough CRPs but is more threatened by machine learning attacks, which makes the PUF-based protocol or system more susceptible. Recent research shows that further investigations are necessary, especially on the concepts of attacks and security analysis.

Recent research shows that the unified design of TRNGs and PUFs is becoming more popular, which decreases consumption and achieves stronger security for authentication. However, no strong PUF unified with TRNG structure has been reported, which could be a promising research field.

We reviewed their designs, the underlying assumptions, and the properties of their implementations. In addition, examples of information security applications, including system security and authentication security, are also presented and discussed.

With this review, we hope that the current spots of entropy harvesting are pointed out.

## Figures and Tables

**Table 1:** The properties of the dynamic/static entropy.

	Dynamic Entropy	Static Entropy
Source	Indeterminate physical process	Manufacturing process
Attribute	Vary with the time	Barely changes over time
Example	Thermal noise, shot noise	Path delay, oscillation frequency

**Table 2:** Performance comparison of TRNG designs.

Design	Technology and Feature	Entropy Type	Throughput (Mbps)	Entropy	Advantage
[58]	200 nm	Noise	4.7	NA	Simple structure
	Oscillator				
	ADC				
[59]	65 nm		3000	0.9996 (max)	High energy efficiency
	Differential amplifier			0.9991 (min)	High speed
[60]	Charge-Trapping FinFET		2000	0.97	Strong robustness
[48]	55 nm	Chaotic	2	0.9997	Low power
	3-T Chaotic Map				
[47]	180 nm		0.27	1	Ultra-low power
	ADC+Chaotic Map				
[49]	FPGA		1600	0.995	High speed
	ACR30				All digital
					Lightweight
[3]	130 nm	Jitter	0.1	0.999	On-chip entropy assurance
	RO				Simple structure
[53]	Artix-7 FPGA		138	NA	Simple structure
	RO				Lightweight
[61]	Zynq-7000 FPGA		12.5	0.999	Lightweight
	Oscillator+TDC				High area efficiency
[57]	Spartan 3E FPGA	Metastability	5	NA	Lightweight
	JK Flip-flop				High area efficiency
	D Flip-flop				Easy integration
[62]	130 nm		2.39	0.9 (min)	Low power
	Noise enhanced latch				Power attack tolerant

**Table 3:** Performance comparison of PUF designs.

Design	Technology	Entropy Type	Inter-Die HD	Intra-Die HD	Advantage
[10]	Virtex4 FPGA	Osillator	46.15%	0.48%	Simple structure
	Digital RO		(1.2 V, 20 ${}^{\circ }$C)	(worst, 1.08 V, 120 ${}^{\circ }$C)	Easy integration
[84]	Current starved RO		49.97%	4.12%	Reliability enhanced
			(1.1 V, 27 ${}^{\circ }$C)	(worst, 0.9 V, 27 ${}^{\circ }$C)	
[85]	General SRAM	SRAM	49.97%	<14%	Easy integration
	FPGA		(nominal FPGA	(worst, −20 ${}^{\circ }$C)	
			power, 25 ${}^{\circ }$C)		
[86]	110 nm		49.10%	5.35%	Better reliability
	SRAM+ECC				
	Linear shift register		(1.5 V, 25 ${}^{\circ }$C)	(worst, 1.5 V, 85 ${}^{\circ }$C)	
[87]	110 nm	Delay	40%	4.82%	Easy integration
	General APUF		(1.8 V, 27 ${}^{\circ }$C)	(worst, 1.85 V, 42.5 ${}^{\circ }$C)	
[88]	65 nm	Voltage	50.26%	4.66%	Low power
			(0.9 V, 27 ${}^{\circ }$C)	(0.9 V, 50 ${}^{\circ }$C)	ML resistant

**Table 4:** Performance comparison of the state-of-the-art TRNG/PUF unified design implementations.

Design	Technology	Entropy Type		Throughput (Mbps)		Robusness	Advantage
		TRNG	PUF	TRNG	PUF		
[5]	65 nm	Electronic noise	Oscillator	100		Injection attack	High speed,
	DAC					& ML attack	Lightweight,
	VCO					resistance	Better Robustness
[113]	28 nm	Jitter based	Current	4.5	12,616	Visual attack	High speed,
	SRAM		mismatch			resistance	High area
	TDC						efficiency
[114]	3D NbOx array	Thermal noise	Current	NA		Injection attack	High reliability
			mismatch			resistance	
[115]	FPGA	Jitter based	Oscillator	12.5		PVT variations	Lightweight,
	DD-cell					resistance	All digital

## Data Availability

Data are available on request.
